# Contrasting patterns in kelp consumption across latitude by two barren forming sea urchin species

**DOI:** 10.1038/s41598-025-33714-z

**Published:** 2026-02-14

**Authors:** Claire Butler, Yanheng Wang, Christopher J. Brown, Adriana Vergés, Scott Ling, Catriona L. Hurd, Scott Bennett

**Affiliations:** 1 Institute for Marine and Antarctic Studies (IMAS), University of Tasmania, Hobart, Tasmania 7001 Australia; 2 Centre for Marine Science and Innovation, School of Biological, Earth and Environmental Sciences, University of New South Wales, Sydney, NSW 2052 Australia

**Keywords:** Macroecology, Ecology, Climate-change ecology

## Abstract

Urchin herbivory is a key function in temperate reef ecosystems. Some urchin species overgraze macroalgal forests, leading to their collapse into barren states. In Australia, climate change is enabling the poleward range extension of urchin species, resulting in increased barrens formation at the cool-edge of their distribution. Despite their ecological importance and association with warming, broad-scale effects of temperature on sea-urchin feeding ecology remain unknown. We characterise in-situ feeding rates of two barrens-forming urchin species, one range-extender (*Centrostephanus rodgersii*), the other range-persistent (*Heliocidaris erythrogramma*), across a temperature range of 8 °C and 12 degrees of latitude, as well as over seasonal cycles. We assess the extent to which ecological drivers (temperature, macroalgal nutrition, urchin size/weight metrics) explain grazing patterns. We find contrasting patterns in, and drivers of, performance between urchin species. *C. rodgersii* shows a peak in grazing and abundance at its range-centre, and temperature is shown to be an important driver of grazing rates for this species. For *H. erythrogramma*, gonad index and macroalgal nutrition are key drivers of grazing rates, which display no significant change across latitude. These contrasting patterns suggest each species occupies different thermal niches, providing key insights into how their ecological impacts may change across their distribution and in response to ocean warming.

## Introduction

Climate change is driving the redistribution of life on earth^1^. As temperatures warm, the cooler extremes of species’ distributions are moving towards higher latitudes, while the warm range-edges are contracting^2^. However, species respond to change in different ways, and key interactions between species may be disrupted or new ones formed, altering the structure and function of ecosystems^3^. Predicting how ecological changes will play out in response to species redistributions is critical for adaptive management and to prioritise efforts in response to climate change. Nevertheless, predicting ecological change is challenging due the multidimensional nature of species responses to warming both between species, and within species, under different levels of organisation, or environmental contexts that manifest across space and time. Uncovering fundamental patterns in key ecological processes through space and time is, therefore, critical for enabling accurate prediction of change.

Herbivory is a critical ecological process that is predicted to change with warming. It plays a key role in the structure and function of ecosystems across terrestrial, freshwater and marine realms^4–7^, and is also tightly coupled to temperature, via temperature’s effect on metabolic rates. Thermal performance theory predicts that as temperatures increase, so do metabolic rates, increasing demand for, and utilisation of, resources^8^. This is particularly true for herbivorous ectotherms whose body temperature and metabolic processes reflect that of the surrounding environment^9^. This has significant implications in context of climate change, and research predicts that herbivore grazing pressure will increase with warming^10–13^.

The relationship between ectotherm physiology and temperature is characterised by a thermal performance curve - a unimodal curve where performance increases slowly with temperature until an optimum temperature, after which performance declines sharply^9,14^.. However, how such relationships manifest across broad-scale environmental gradients is poorly resolved. Similarly, how physiological performance translates to ecological processes across environmental gradients is unknown for most species. For herbivory, spatial and temporal variation in plant nutritional quality can influence grazing rates, potentially dampening or exacerbating temperature induced effects on feeding. Low nutrient conditions, for example, can lead to compensatory (i.e. higher) feeding rates by herbivores to fulfil metabolic demands^15,16^. Similarly, spatial variation in herbivore size (e.g. in response to differences in population age structure, fishing pressure or food availability) may change feeding behaviour. Larger herbivores may consume more than smaller herbivores, although have lower mass-specific grazing rates^17^. Across temporal gradients, seasonal variation in reproductive investment may either increase energetic needs and/or hinder digestion influencing feeding rates^18^.

Species’ life history traits also have important implications for the physiological performance and potential ecological impact of different populations of a species across broad scales. For species with high dispersal capacity and high connectivity between populations, we might expect them to show a conserved thermal niche - that is low variation in thermal performance among individuals but strong differentiation in performance across a species range in response to environmental gradients in temperature [e.g^19^.. Conversely, for species with low dispersal capacity and low connectivity, isolation can drive local adaptations in thermal performance, dampening differences in performance between populations, when observed across broad geographical gradients in temperature [e.g^20^.. An understanding of how contrasting life history traits may impact ecological processes like herbivory across broad spatial scales is therefore important.

Kelp forest ecosystems are well suited for understanding the impact of climate-driven changes in herbivore pressure. Kelp forests are among the most extensive, productive, and biodiverse coastal habitats, globally^21–24^. However, kelp forests are at risk of herbivore-driven ecosystem collapse^25,26^. An understanding of climate impacts on the grazing rates of dominant herbivores can therefore vastly improve our understanding of potential impacts of climate change across these important ecosystems.

In Australia, kelp forests span over 8,000 km of the southern coastline and are host to two dominant sea urchin herbivores. The long-spined and short-spined urchins (*Centrostephanus rodgersii* and *Heliocidaris erythrogramma* respectively) both have the capacity to catastrophically overgraze kelp forest habitats, forming barren grounds devoid of canopy-forming macroalgae^27–29^, but contrast in their thermal affinities and life history traits. *Centrostephanus rodgersii* is a warm affinity species, with a historical distribution extending from southern Queensland to Bass Strait on the east coast of Australia^30^. However, over the last 4 decades this species has extended its range into Tasmania, where it has now established significant populations^27^. This range extension has been facilitated by *C. rodgersii’s*long larval phase (6 - 16 weeks^31,32^;) which enables poleward transport via the East Australian Current. In contrast, *H. erythrogramma* is distributed across the entire temperate Australian coastline and its distribution has remained fixed with no apparent evidence indicating climate-driven shift in recent decades. *Heliocidaris erythrogramma* has a cooler affinity and a comparatively short larval phase lasting just 3-5 days^33^.

In addition to differing biological traits, the ecological impact of these two urchins on kelp forest ecosystems is vastly different. *Heliocidaris erythrogramma* is known to form barrens, however, these are locally restricted to wave-sheltered reef areas where supply of macroalgal drift becomes limiting^28,34^. *Centrostephanus rodgersii* in contrast, forms extensive and widespread barrens across its entire range^25,30^. The current extent of *C. rodgersii *barrens increases from warm- to mid-latitudes in the northern half of its range, with the most extensive barrens in this region typically found from 36-37°S^35^. In the cool-edge of its range in Tasmania, the extent of barrens is on the rise, with an estimated 15% loss of shallow kelp habitat since the urchin was first observed in 1970’s^27^. Given that these species coexist in an ocean warming hotspot, where ocean temperatures are increasing at four times the global average^36^, an understanding of how warming will influence their grazing impact across their range is required.

Here we examine spatial and temporal patterns in *in-situ* grazing performance of two barren-forming urchin species (*C. rodgersii* and *H. erythrogramma*). Grazing performance is assessed across a steep climatic gradient spanning their latitudinal distribution, as well as seasonally across an 18 month period in a single high-latitude location. Grazing rates are related to ecological variables (namely temperature, kelp nutritional value, and urchin reproductive status) to assess their importance in driving patterns in grazing performance through space and time. Ultimately we seek to gain an understanding of how temperature influences the grazing impact of these dominant and functionally important herbivores on temperate reef ecosystems across their range, and the implications this has in context of continued ocean warming.

## Methods

### Study sites

**Fig. 1:**
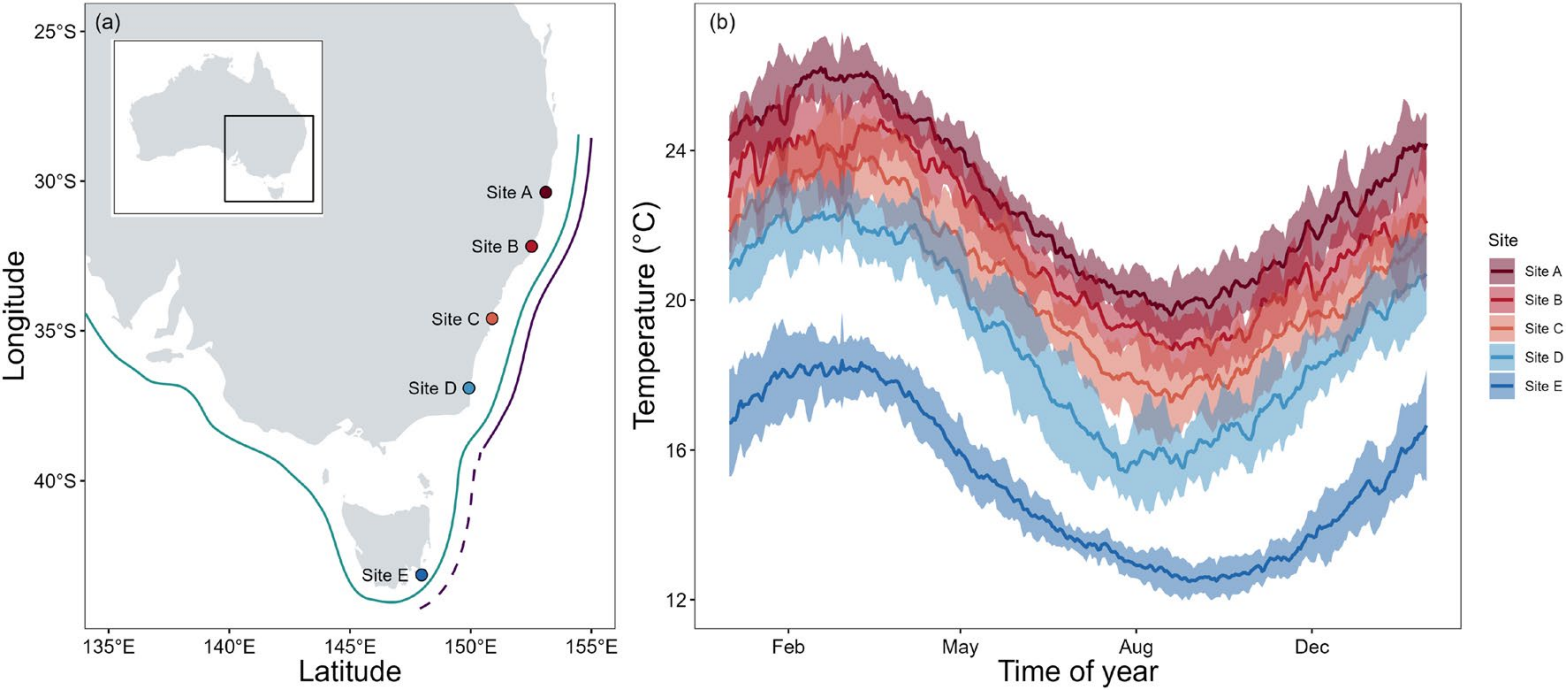
(**a**) Map of experimental sites A-E showing the natural (solid line) and extended (dashed line) distribution of *C. rodgersii* (purple) and *H. erythrogramma* (green). (**b**) seasonal temperature profiles at each site based on mean daily temperatures averaged for the period 2012-2023. Shaded areas represent the standard deviation in daily temperatures between years. Map was generated using R^38^.

Sampling was undertaken across five sites spanning the latitudinal range of both sea urchin species in southeast coast of Australia (Fig. 1). Coastal thermal regimes in this region are dominated by the East Australian Current, a warm water current that flows from north to south along Australia’s east coast and gives rise to a strong latitudinal gradient in temperature^37^. The five sites – Site A (Sawtell; -30.38, 153.11), Site B (Forster; -32.18, 152.52), Site C (Shellharbour; -34.59, 150.89), Site D (Merimbula; -36.90, 149.93) and Site E (Fortescue Bay; -43.14, 147.97) - were chosen such that they broadly spanned both species range and thus thermal distribution in Australia, and, where possible, were spaced at regular intervals relative to the mean annual sea surface temperature (SST) (typically 1-1.5 °C between sites). Local site selection also took into account the comparability of habitat types. Sites were chosen on exposed to semi-exposed sections of open rocky coastline with medium to dense cover of canopy-forming species and where *Ecklonia radiata* constituted a dominant species (for descriptions of each site see Table S1). Each of the five sites was visited once over a 7-week period in February/March 2023. Sequential sampling from highest to lowest latitudes was performed for logistical reasons. Sequential sampling of sites minimised the difference in temperature between sites but ensured sampling occurred at peak summer ocean temperatures in each location. For *C. rodgersii*, assays were not completed at site B due to a lack of shore-accessible urchins.

Seasonal field sampling was undertaken at the southern-most site only, site E (Fortescue Bay), on the southeast coast of Tasmania (Fig. 1). Assays were carried out every 1-3 months between September 2021 and March 2023, with a total of 14 sampling trips occurring over the 19-month period.

### Temperature data

Mean daily satellite-derived SST data for each site were extracted from the NOAA National Climate Data Centre Optimally Interpolated AVHRR product at a 1/4° grid resolution (Optimum Interpolation Sea Surface Temperature, www. ncdc.noaa.gov/oisst).

Underwater temperature loggers (ONSET HOBO MX2201, Onset Computer Corp., Bourne, MA, USA) were also deployed at each site/sampling period for the duration of each grazing assay. For two sites across the latitudinal analysis where these loggers flooded (Site B, D), *in-situ* temperatures were estimated using dive computer records (Suunto Zoop) during deployment and retrieval.

### Grazing assays

*In-situ* grazing assays were carried out at each site/sampling period. For each assay, 30 individual *C. rodgersii* and 30 individual *H. erythrogramma* were collected by hand from 2-10 m depths and in close proximity to kelp habitat. *H. erythrogramma* were typically collected from 2-5 m depths, while *C. rodgersii* were found slightly deeper from 2-10 m depths. Urchins with a test diameter over 80 mm were targeted at each site/sampling period, however in locations were this size class was not found, the urchins with the largest test diameter available where collected (see Figs. S1, S2, Table S2 for size distributions at each site/sampling period). Urchins were haphazardly placed inside one of 12 individual compartments (265 mm x 235 mm x 110 mm) in one of 6 weighted inclusion cages. Inclusion cages were constructed of a plastic frame with 1cm aperture plastic mesh coating, including for compartment dividers. Any given cage would contain 5 urchins of each species in a separate compartment, and two urchin-free compartments acting as controls to standardise the autogenic weight change due to kelp growth or erosion. Urchins were kept entirely submerged throughout the whole process to minimise stress.

Urchins were fed fresh lateral blades of the kelp *Ecklonia radiata*, collected on scuba at the same site and time of deployment. *Ecklonia radiata* is the dominant kelp in temperate Australia and a key diet item of both urchin species^39–41^. For latitudinal assays whole or partial lateral blades were torn from adult sporophytes, while for seasonal assays blades were cut into standardised 30 mm x 8 mm sections using a cookie-cutter and urchins fed six sections each (approximately 10 g). In both cases only healthy blades free of epiphytes and erosion were selected from the younger half of the plant. Blade sections were chosen at random and were blotted dry using paper towel. An initial wet weight was recorded to the nearest 0.00 g before the pieces were fastened to the base of each compartment using cable ties. The placement of kelp in this way ensured it was easily accessible to the sea urchins. An image of a grazing cage after collection is provided in Fig. S3. Kelp was kept submerged and shaded before and after weighing to avoid desiccation and heat-stress.

Once urchins and/or kelp blades were placed in each compartment and the inclusion cages closed and secured, cages were left on the seafloor for up to 8 days. For seasonal assays, deployment duration ranged from 5-8 days, while for latitudinal deployments the duration was 3-7 days. During deployments, cages effectively isolated urchins from access to additional food sources. Upon collection urchins were checked for any signs of ill-health (drooping or unresponsive spines, physical damage), and, given good health, the remaining kelp in each compartment was blotted dry with paper towel and weighed on site. Blotting was carried out by the same person before and after any given deployment for consistency. Individual grazing rates were calculated as:

$$\frac{\Delta WW_a - \overline{\Delta WW_c}}{No.days}$$

where $WW_a$ is the wet weight of the assay and $WW_c$ is the wet weight of the control. For seasonal assays, remaining kelp from every compartment was bagged individually and dried in an oven at 60 °C until dry weights were constant. Dry weights were measured to validate the consistency of the blot dry method.

### Urchin size and weight metrics

Upon completion of grazing assays, all urchins were transported back to the lab (either nearby accommodation for latitudinal fieldwork, or for seasonal fieldwork to the Institute of Marine and Antarctic Studies, University of Tasmania), to collect data on size and weight metrics. Measurements were collected the day of (latitudinal) or day following (seasonal) assay completion. Measurements of test diameter, test height and whole wet weight (includes coelomic fluid) were recorded, and the urchin then dissected to collect test weight, lantern weight, gonad weight and gut contents weight. Gut contents were drained through a 1 mm sieve before measuring to minimise water weight. Gut contents were dried in an oven at 60°C until weights were constant, and dry weight was recorded to validate the consistency of the draining method. These size and weight metrics were used to calculate the following urchin body metrics:

$$\text {Drained weight} = \text {Test weight} + \text {Gonad weight} + \text {Gut weight} + \text {Lantern weight}$$

$$\text {Gonad index} = \Biggl (\frac{\text {Gonad weight}}{\text {Whole wet weight}}\Biggl )*100$$

$$\text {Gut index} = \Biggl (\frac{\text {Gut weight}}{\text {Whole wet weight}}\Biggl )*100$$

The drained weight differs from the whole wet weight in that is does not include coelomic fluid.

### Urchin abundance

Time-averaged abundance of each urchin species within the study region was estimated using the Reef Life Survey (RLS) database. RLS surveys have been carried out at hundreds of locations across southeast Australia since 1992, and provide high resolution data of abundance across latitude^42,43^.

### Kelp carbon and nitrogen content

Macroalgal samples were collected from each site and sampling period for analysis of carbon and nitrogen content. This was used to assess any variation in nutritional quality of *E. radiata* between sites^44^. For latitudinal analysis, samples were kept in fresh seawater and frozen at -20°C within 4 hrs of collection before being freeze-dried at the end of the 7-week fieldtrip. For the seasonal analysis samples were kept cool, damp and dark for up to 4 hours until being placed in a drying oven at 60 °C until weights stabilised. Dried and freeze-dried samples were finally ground to a homogenous powder using a mortar and pestle.

Tissue carbon and nitrogen content (% dry weight) was determined by weighing $\sim$2 mg of dried sample into tin cups for analysis using flash combustion isotopic ratio mass spectrometry (varioPYRO cube coupled to an Isoprime100 mass spectrometer) at the Central Science Laboratory, University of Tasmania, Australia. International reference standards were used to correct for instrumental drift and quality assurance, and performance of the instrumentation, drift correction and linearity performance were calculated from repetitive analysis of these standards. Precision was 0.1 ‰.

### Data analysis

#### Temperature regimes

We used a generalised additive mixed model (GAMM) to test for differences in annual climatology between each of the study sites across latitude. GAMMs use splines to identify smooth non-linear relationships between the response and predictor variable^45^. The GAMM included a fixed effect for site, and a spline for day of year, as well as a random effect spline for year to account for year-to-year variation. Inspection of ACF correlation plots indicated substantial autocorrelation in the residuals, and to accommodate this we used an AR(1) structure.

#### Grazing rates models

We used generalised additive models (GAM) with a gaussian family to examine if there were any consistent patterns in grazing rates across latitudinal and seasonal gradients. Upon inspection of diagnostic plots, clear heteroscedastic trends in the residuals of each predictor against latitude and season were evident for both *C. rodgersii* and *H. erythrogramma*. To accommodate this, we fit GAMs using the gaussian location-scale family (GAMLSS). The gaussian location-scale model is a multi-predictor model with explanatory variables for the mean and the standard deviation^46,47^. In this case we included latitude (for latitudinal data set) and time (for seasonal data set) as predictors for the standard deviation, for both species.

Due to the nature of the data collection, approximately 20% of both the latitudinal and seasonal grazing rates data sets were slightly negative after accounting for controls. These negative values may either represent net biomass gain (i.e. growth) of kelp despite grazing activity, or in some cases may also result from measurement error when measuring wet weight before and after deployment, particularly in instances when urchin did not graze or grazed very little. High variability in grazing rates, including low or zero values, is common in herbivory experiments across broad spatio-temporal scales^48,49^. Removing or zero-ing these negative values would artificially change the error estimation in statistical analyses and for this reason, the negative values were left unchanged for analysis. To ensure this decision did not influence model results, we also fit a GAMs with a tweedie distribution to data where negative values were zero-ed. These models did not change observed patterns through space/time and are presented in the supplementary material only (Figs. S4, S5, S6, S7, Tables S3, S4, S5, S6).

Urchin body size is an important factor that influences metabolic rates and can impact grazing rates^8,17^. Urchin body size can also vary between populations and through time within populations due to factors such as population age structure, fishing pressure, recruitment success, or food availability^50–52^. A robust understanding of how body size influences grazing rates was beyond the scope of this study. However, to account for body size differences both within and between sites and sampling periods, we used mass-independent consumption rates in all analyses. Mass-independent consumption was calculated by regressing absolute consumption rates on log-transformed whole wet weight and extracting the residuals^52^. We selected whole wet weight to represent urchin size because this is an easily obtained and commonly used metric, and highly correlated to test diameter. To ensure this approach did not influence our results we also modelled mass-specific grazing rates across latitude that included whole wet weight as a predictor. These models showed similar results to the mass-independent models and are presented in the supplementary material (Figs. S8, S9, S10, S11, Tables S7, S8, S9, S10).

**Latitudinal grazing rates models**

For assessing grazing rates across latitude, mass-independent consumption was modelled against gonad index, gut index, and latitude. Gonad index was included in the model as a proxy for reproductive status of each individual. *Centrostephanus rodgersii* in particular is known to show clear trends in gonad index throughout a reproductive cycle, with lowest indices in October immediately after spawning and increasing through summer to a pre-spawning peak in June^53^. Gut index also shows seasonal trends, possibly related to gonad index^53^, and due to long gut passage times (> 3 weeks; pers. obs) provides a measure that reflects initial condition of the urchin upon collection. In addition, how ‘full’ the urchin test is may also influence its feeding rate because there is only finite space inside the test. All models included cage deployment ID as a random effect smooth. Compartment ID was not considered in the model because the haphazard placement of cages meant that individual compartments were not subject to the same conditions within each deployment, depending on current/swell strength and direction and the cage position relative to reef topography.

**Seasonal grazing rates models**

For seasonal grazing rates, the larger number of sampling events (14 time points relative to the 5 latitudinal sites) allowed for a more robust statistical assessment of the importance of different environmental variables in driving grazing rates. In addition to variables included for latitudinal models (gonad and gut index, cage deployment ID), seasonal models also included the duration of deployment, the mean temperature during assay deployment, mean % nitrogen of *E. radiata*, and the number of daylight hours [calculated for Fortescue Bay using the *daylength()* function from the *geosphere* package in R^54^). These latter three additional variables are the key parameters that change across seasons which may influence urchin grazing rates. Because temperature, nitrogen and day length were highly correlated, we compared selected models that included different combinations of these variables to test if they contributed significantly to explaining any variation in grazing rates^55,56^. Similarly, because the growth and maintenance of gonad requires significant energy input, we further tested interactions between gonad index with temperature and nitrogen. Final model selection was based on AIC (Tredennick et al. 2021^57^). For *C. rodgersii*, several models presented an AIC within 2.5 of the lowest AIC, and here the simplest model was selected (and all similar models presented in supplementary material; Figs. S12, S13, Tables S11, S12). Similar to latitudinal models, seasonal models were fitted using mass-independent grazing rates to account for urchin size differences between sampling events.

#### Kelp C:N, urchin abundance and urchin size models

Models to asses latitudinal and seasonal patterns in kelp carbon and nitrogen content were constructed as single term GAMs. For urchin abundance across latitude, separate models were fit for each species using a negative binomial GLM. For *C. rodgersii* a second order polynomial on latitude was included. Differences in urchin size between between sites across latitude and between temporal sampling events were assessed using ANOVA. Pairwise comparisons were made using Tukey HSD.

All models were inspected to ensure they met model assumptions of homogeneity of variances and normality of residuals and transformations used when applicable (see Table S13 for a summary of each model, including any applied transformations). For predictions, the *predict()* function relevant to the specific model structure was used to calculate model predicted means and standard errors (i.e. *predict.gam(), predict.glm*). All analyses were conducted in R^38^. GAMs were fit using the ‘mgcv’ package^45,47^, and GLMs were fit using ‘MASS’^58^.

#### Urchin grazing pressure model

Urchin grazing pressure across latitude was estimated by multiplying predictions of mean mass-independent grazing rate and urchin abundance across latitude. Uncertainty was calculated by simultaneously running a non-parametric bootstrap for both models, then multiplying the resulting grazing rate and abundance estimates for each site. Thus the final calculation incorporates uncertainty in both grazing rates and abundance for each species^59^.

## Results

### Temperature regimes at experimental sites

Study sites, which spanned 12°of latitude, differed in mean daily sea surface temperatures by 8°C. At the warm-edge of the gradient, annual temperature profiles at experimental sites ranged from 19.6 ± 0.22°C to 26.2± 0.23°C in site A (mean ± SE from 2012 to 2023), 18.5 ± 0.21°C to 24.8 ± 0.26°C at site B and 17.3 ± 0.33°C to 24.2 ± 0.38°C at site C (Fig. 1). At the cooler edge of the gradient, annual mean temperatures ranged from 15.4 ± 0.23°C to 22.6 ± 0.27°C at site D and 12.5 ± 0.14°C to 18.4 ± 0.75°C at site E (Fig. 1). Modelling revealed different annual SST climatologies between sites. Site means and standard errors are shown the supplementary material (Figs. S14, Table S14).

**Fig. 2:**
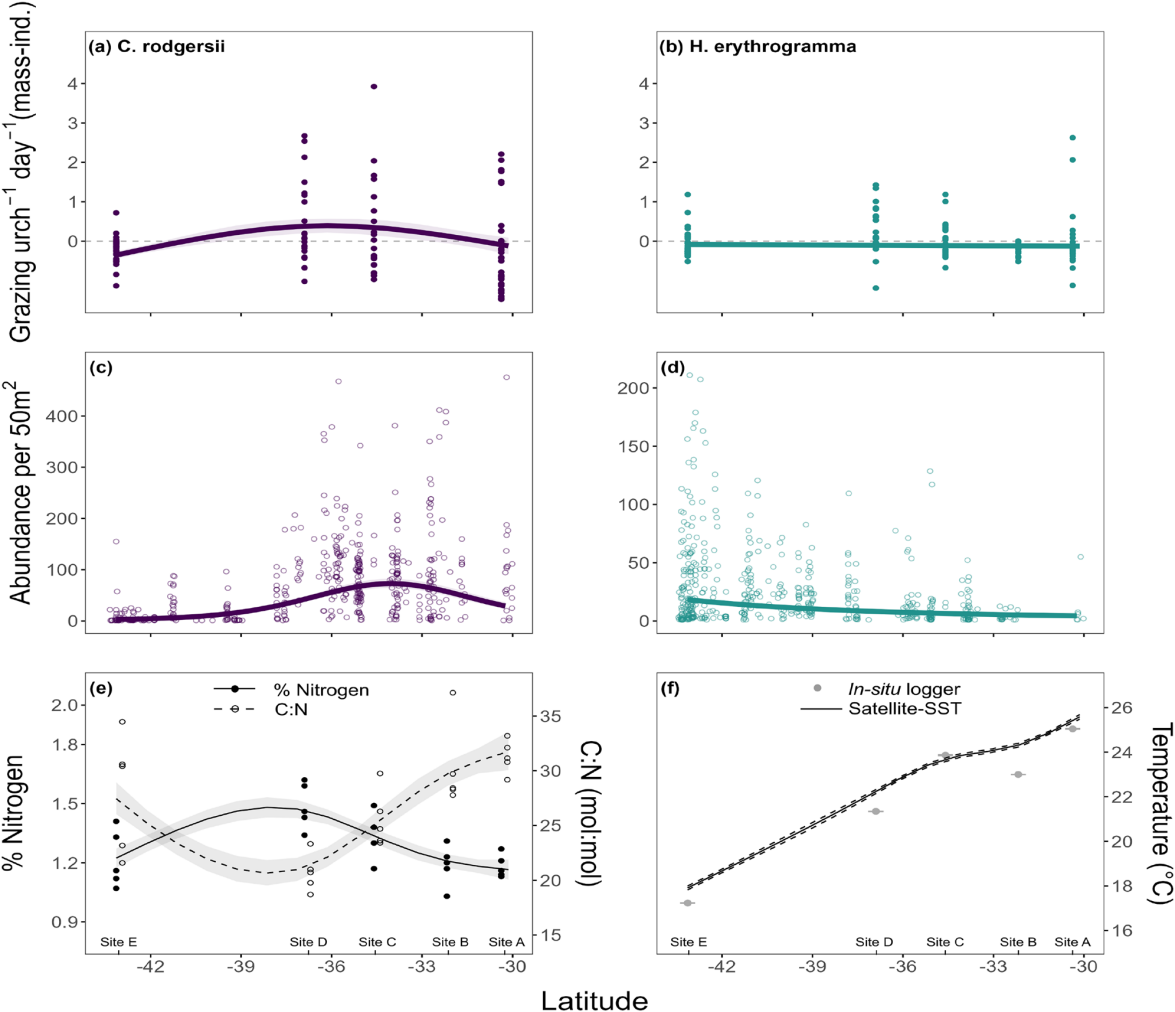
Mass-independent grazing rates across latitude for (**a**) *C. rodgersii* and (**b**) *H. erythrogramma*, where points represent raw residuals from rate-mass regression and the line shows the predicted trend in grazing rates (mean ± se). For *C. rodgersii*, assays were not completed at site B due to an unexpected lack of shore-accessible urchins. Mean abundance of (**c**) *C. rodgersii* and (**d**) *H. erythrogramma* as recorded on RLS surveys across the latitudinal range of the study area. Points represent time-averaged abundance at different sites while the solid line shows the model predicted mean ± se across latitude. Panel (**e**) shows % nitrogen content (solid line and solid points) and C:N ratio (mol:mol; dashed line and hollow points) of *E. radiata* and panel (**f**) shows two different estimates of temperature. The grey points show the mean temperature that was recorded over the duration of the grazing assay using *in-situ* temperature loggers, while the solid black line shows the model-predicted mean temperature across latitude based on satellite-derived daily SST means from January, February and March of 2023 (the sampling period) at each site. The dashed lines represent the standard error.

Latitudinal fieldwork was carried out over summer, with in-situ temperatures at each site all above the 74^th^ percentile of annual temperature profiles, except site B, where *in-situ* temperature was at the 67^th^ percentile of the local climatology. *In-situ* temperature at site B (23°C) was lower than at its cooler-latitude neighbour, site C (23.9 °C; Fig. 2f). When compared to average temperature observed at this site over February in the last 10 years, the mean temperature we recorded at site B was 1.1°C below average, while site C was 0.3°C above average (Fig. 2f).

Seasonal variation in temperature is similar at each site, with coolest temperatures typically occurring in late winter and early spring (Aug/Sept) and warmest temperatures in late summer and early autumn (Feb/March; Fig. 1). *In-situ* temperatures recorded during grazing assay deployment reflected this, with peak temperatures recorded in summer between January and April each year (Fig. 4f). Highest average temperatures across these four months ranged from 17.23 - 18.04°C, whilst lowest average temperatures ranging from 12.4 - 12.9°C were recorded from June to October each year.

### Latitudinal patterns

#### Latitudinal patterns in grazing rates

Overall, *C. rodgersii* consumed more kelp than *H. erythrogramma*, with mean consumption rates ranging from 0.41 ± 0.6 to 1.23 ± 0.23 g urchin^−1^ day^−1^ (mean ± SE) across all sites, compared to 0.07 ± 0.03 to 0.49 ± 0.13 g urchin^−1^ day^−1^ for *H. erythrogramma*.

Grazing rates for *C. rodgersii* and *H. erythrogramma* showed clear differences across latitude. Generalised additive models revealed significant trends in grazing rates across latitude for *C. rodgersii*, but not for *H. erythrogramma* (Figs. 2a,b, S15, S16, Tables S15, S16). *C. rodgersii* grazing demonstrated a hump-shaped pattern across latitude, with highest grazing rates recorded at centre-range sites between approximately 38-34°S, and decreased rates observed at both the warm and cool range edges (Fig. 2a). In addition to latitude, gut index and variance with latitude had a significant effect on *C. rodgersii* grazing rates (Table S15). The model summary (Table S15) and partial effects plots of this model (Fig. S15), show a significant and positive linear effect of gut index on grazing rates, as well as a significant non-linear increase in the variability of grazing rates with decreasing latitude. Gonad index and cage ID did not have a significant effect. This model explained 27.4 % of deviance.

For *H. erythrogramma*, no significant effect of latitude on grazing rates was found (Table S16). Grazing rates of *H. erythrogramma* were significantly affected by gut index, while all other model terms were insignificant (Table S16). This model explained 32.1 % of deviance. The partial effects plots of this model (Fig. S16), show a significant and positive linear effect of gut index on grazing rates.

#### Latitudinal patterns in urchin abundance

Urchin abundance varied between species and between sites across latitude. For *C. rodgersii* abundance demonstrated a non-linear trend with a single peak in central sites, between approximately 33-36°S (Fig. 2c). Urchin abundance in this region (sites C, D) reaches up to 67.3 ± 6.4 urchins per 50 m^2^ (mean ± se), and drops to 19.1 ± 3.1 urchins per 50 m^2^ at the warm range edge and 2.8 ± 0.3 urchins per 50 m^2^ at the cool-edge. This peak in abundance broadly coincides with the peak in grazing rates between 33-39°S.

Abundances of *H. erythrogramma* show a non-linear trend with highest abundances observed at the cool range edge (Fig. 2d). Here, mean abundance was 21.3 ± 2.5 urchins per 50 m^2^, while at the warm-edge mean abundance was 3.3 ± 0.2 urchins per 50 m^2^.

#### Urchin size across latitude

Whole weight of *C. rodgersii* varied between 163-645 g across all sites (Fig. S1, Table S2). There was a significant effect of site on mean whole wet weight (ANOVA: F_3, 103_ = 31.6, p < 0.000, Table S17) and pairwise comparisons reveal Site E and site C to have similar mean whole weights while all other pairwise combinations were significantly different to each other (Fig. S17).

For *H. erythrogramma*, whole weights ranged from 16-503 g across all sites (Fig. S1, Table S2). Site significantly effected whole wet weight (ANOVA: F_4, 115_ = 39.85, p < 0.000, Table S18) with sites A, C and D showing similar mean whole weights, as well as site A and B (Fig. S18).

#### Latitudinal patterns in kelp carbon and nitrogen content

Nitrogen content of *E. radiata* increased with latitude until approximately 38°S, however nitrogen content of kelp at the single site south of this was relatively low (Fig. 2e). The highest mean kelp nitrogen content was observed at site D (centre-range, 1.49 ± 0.05 se) with the mean nitrogen content dropping to 1.18 ± 0.3 se to the north and 1.22 ± 0.06 se to the south.

Mean C:N ratio (± se) varied from 20.7 ± 1.7 to 31.3 ± 1.5 (mol:mol) across latitude, and followed the opposite pattern to nitrogen; when nitrogen content was high, the C:N ratio was low and vice versa (Fig.2e). The highest C:N ratios were observed at warm and cool range edge locations (sites A, B, E; mean range 28-31.3:1), while central site D recorded the lowest ratio (20.7:1).

#### Latitudinal patterns in urchin grazing pressure

**Fig. 3:**
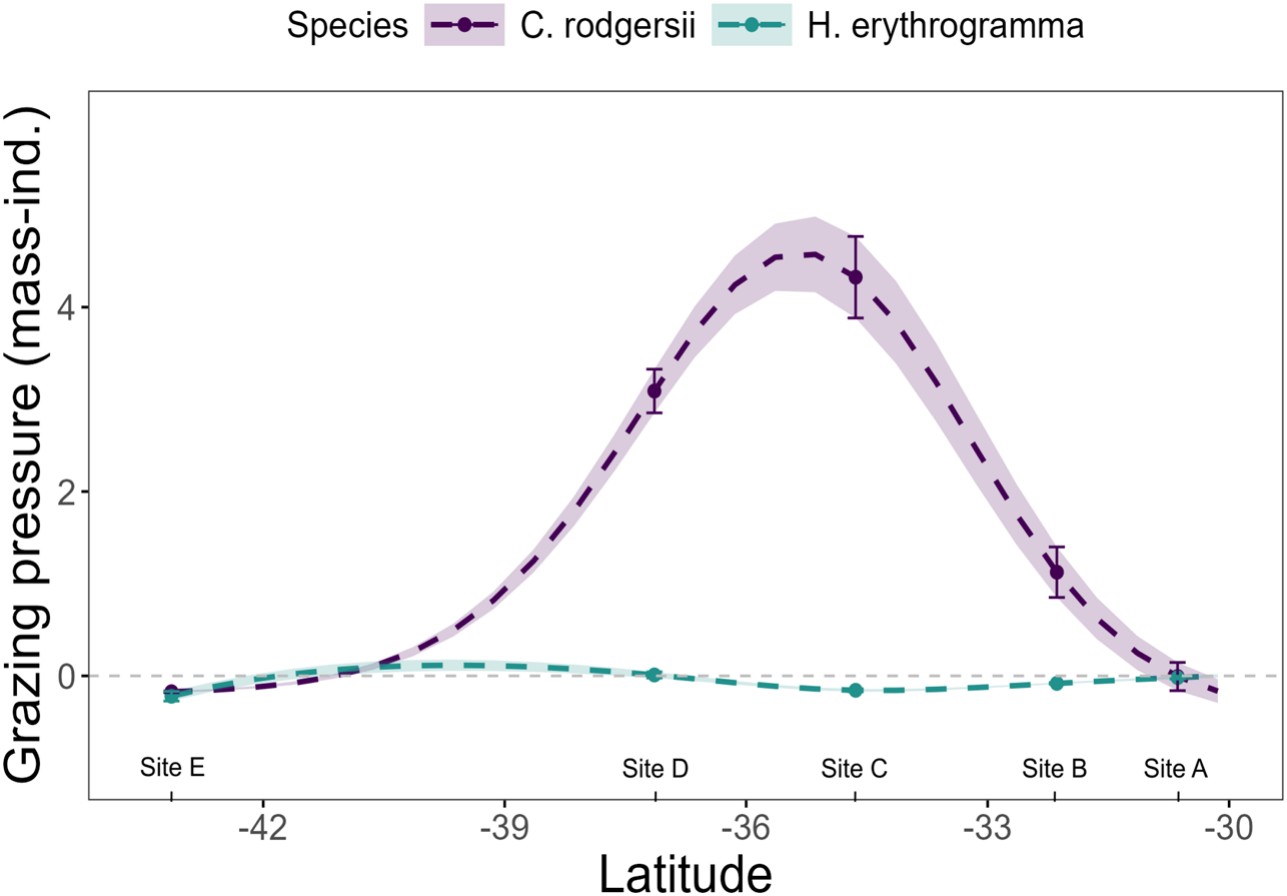
Mass-independent grazing pressure of *C. rodgersii* and *H. erythrogramma* as estimated using the mean mass-independent grazing rates and RLS urchin abundance across latitude. Dashed lines and shading represents mean ± se, and points with error bars highlight site locations.

Grazing pressure varied between sites and between species across latitude. For *C. rodgersii* grazing pressure followed a pattern that reflected the overlapping peaks of grazing rates and abundance counts, with a clear peak in grazing pressure observed at -35.5°S (between central sites C and D; Fig. 3). Grazing pressure for *H. erythrogramma* was comparatively low and constant across latitude (Fig. 3),

### Seasonal patterns

#### Seasonal patterns in grazing rates

**Fig. 4:**
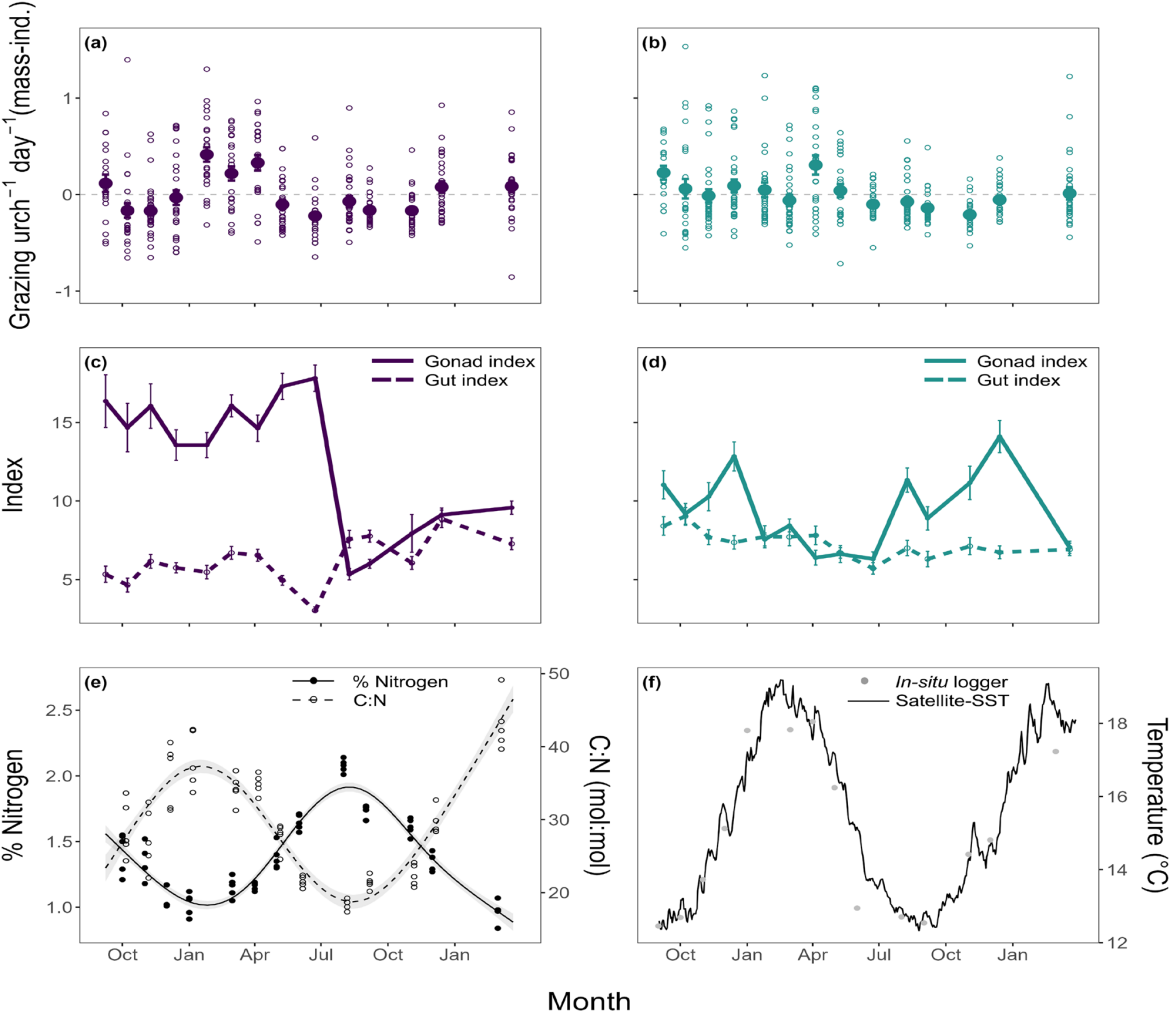
Mass-independent grazing rates for (**a**) *C. rodgersii* and (**b**) *H. erythrogramma* through time, where open-circles represent raw residuals from rate-mass regression and solid points shows the mean (± se) value for each time point. Panels (**c**) and (**d**) show gonad (solid line) and gut indices (dashed line) for *C. rodgersii* and *H. erythrogramma* respectively (mean ± SE). (**e**) Percentage nitrogen content (solid line and solid points) and C:N ratio (mol:mol; dashed line and hollow points) of *E. radiata* through time and (**f**) temperature as recorded at 3-7 m depths by *in-situ* loggers deployed over duration of grazing assays (grey points; mean ± SE) and daily averages from satellite SST (black line).

Grazing of *C. rodgersii* and *H. erythrogramma* varied through time, with higher rates observed over summer months, particularly for *C. rodgersii* (Fig. 4). Similar to latitudinal patterns, *C. rodgersii* generally consumed more kelp than *H. erythrogramma*, with mean consumption rates ranging from 0.13 ± 0.04 to 0.77 ± 0.08 g ind^−1^ day^−1^ across all time points, compared to 0.02 ± 0.12 to 0.68 ± 0.1 g ind^−1^ day^−1^ for *H. erythrogramma*).

**Fig. 5:**
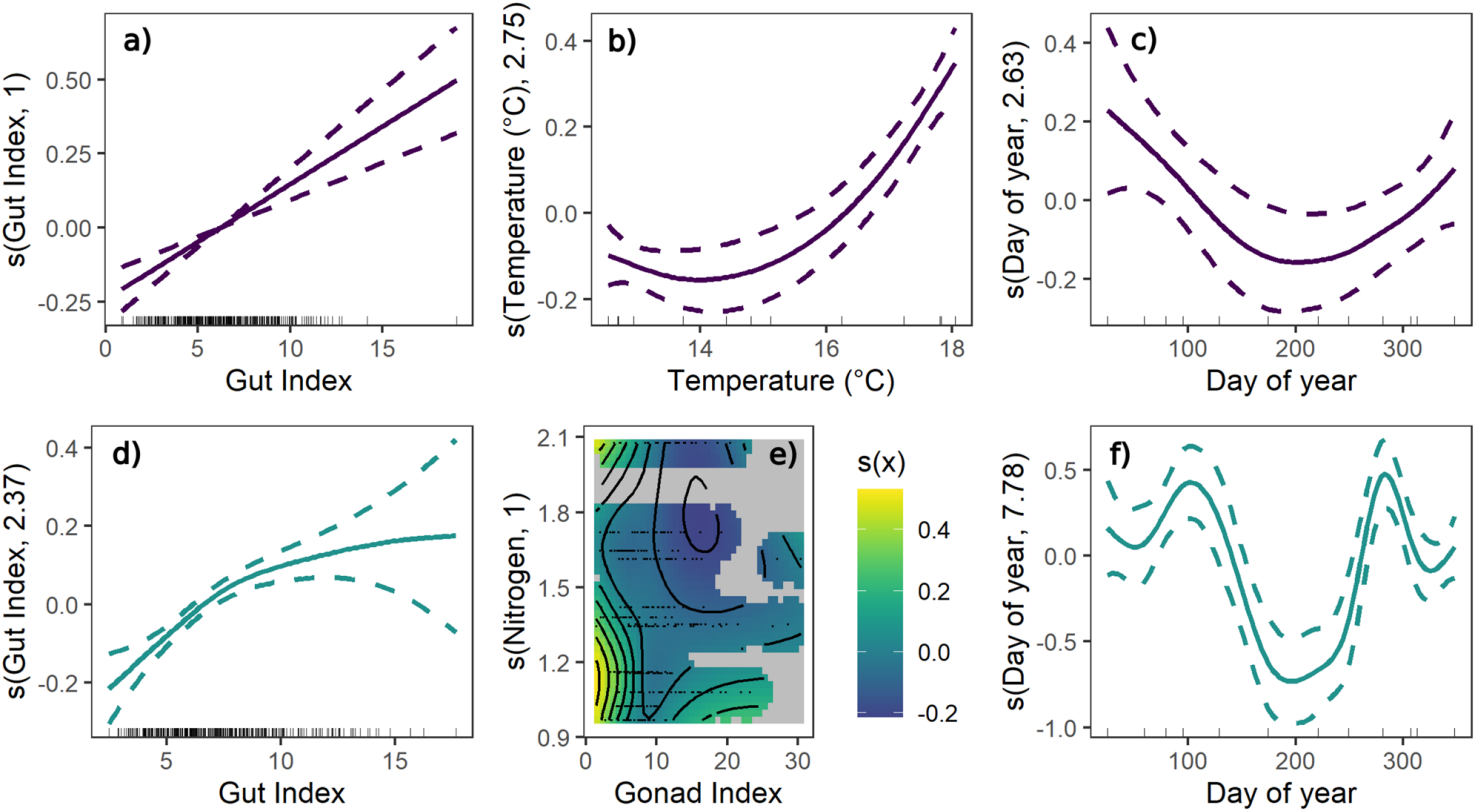
Partial effects plots of selected variables with significant effects from the best-fit GAMs for mass-independent grazing rates of the sea urchins *C. rodgersii* (**a**–**c**, purple) and *H. erythrogramma* (**d**–**f**, green). These plots show the estimated effect of the predictor variable on grazing rates when all other model variables are held constant (centred around 0). The y-axis shows the contribution of the predictor to the additive component of the model, and does not represent the response variable directly. Negative values indicate where the effect of the predictor reduces grazing rates below average, and positive values indicate where the predictor increases grazing rates above average. Solid and dashed lines represent the mean ± 95% CI. The heat map in (**e**) shows the predicted value of the smooth term *s(x)* (i.e. the contribution to the additive component of the model) for the interaction between kelp nitrogen content and gonad index for *H. erythrogramma*.

Models revealed contrasting drivers of grazing rates through time for *C. rodgersii* and *H. erythrogramma*. For *C. rodgersii*, multiple models displayed similar AIC values (within 2.5 units of the model with lowest AIC; Table S19). The simplest of these models included all main effects (gonad index, gut index, duration of deployment, mean temperature, day length) except for nitrogen (Fig. 5, S19, Table S20). It shows a significant and positive effect of gut index and temperature on grazing rates (Figs. 5, S19, Table S20). Assay duration, gonad index, day length and cage ID do not significantly affect grazing rates, while the variance in grazing rates through time shows a significantly non-linear trend with larger variance over summer months (Figs. 5, S19, Table S20). This model explains 33.0% of deviance. Other models with similar AIC included either a significant interaction between gonad index and nitrogen or temperature and nitrogen (Table S19). Interpretation of drivers differs between models, however common to each model, is a significant positive effect of gut index and temperature on grazing rates (Figs. S12, S13, Tables S11, S12).

For *H. erythrogramma*, the best-fit model included main effects of gut index, mean temperature and duration of assay, and an interaction between gonad index and nitrogen (Fig. S20, Table S21). Gut index had a significant and positive effect on grazing rates, while the interaction shows that *H. erythrogramma* consume more when their gonad index is low, regardless of the nitrogen content of *E. radiata*. Temperature, assay duration and cage ID had no significant impact on grazing rates for *H. erythrogramma*, and the variance in grazing rates through time shows a significant non-linear trend with larger variance in late spring and summer (Fig. S20, Table S21). This model explained 32.5% of deviance.

#### Seasonal patterns in kelp carbon and nitrogen

Mean nitrogen content of kelp tissue ranged from 0.97 ± 0.04 to 2.08 ± 0.02 % over the 19-month period. Nitrogen content followed a pattern that mirrored SST, with highest nitrogen content observed over winter when temperatures were at their lowest, and lowest nitrogen recorded over the summer months (Fig. 4e).

Mean C:N ratio varied from 18.4 ± 0.7 to 43 ± 1.6 (mol:mol) over the 19-month period. C:N followed an opposite pattern to nitrogen, with the highest ratios observed over summer months when nitrogen was lowest and vice versa.

#### Seasonal size and weight metrics

Whole weight of *C. rodgersii* ranged from 87-559 g across the sampling events, while for *H. erythrogramma* sizes varied from 66-538 g (Fig. S2). There was a significant effect of sampling event on whole weight for *C. rodgersii* (ANOVA : F_12, 352_ = 4.72, p < 0.000, Table S22) and *H. erythrogramma* (ANOVA : F_12, 350_ = 3.17, p = 0.001, Table S23). Pairwise comparisons are shown in the Supplementary Figs. S21, S22.

For both *C. rodgersii* and *H. erythrogramma*, gonad index varied between months. Gonad index showed an overall increase in the months prior to spawning, and spawning is evidenced by a sharp decrease in gonad index over winter ($\sim$ August) for *C. rodgersii* and over summer ($\sim$ December-January) for *H. erythrogramma* (Fig. 4c, d). For *H. erythrogramma* gonad index recovered to similar levels after each of the two observed spawning events. For *C. rodgersii*, where only one spawning event was recorded in the time period, gonad index does not recover to pre-spawning levels before the end of the sampling observations. Gut index for both species showed no clear seasonal pattern, although for *C. rodgersii* there is a decrease in gut index in the months leading up to spawning. This is reflective of the period of resource acquirement and subsequent transfer of energy to gonad production before spawning.

## Discussion

### Contrasting latitudinal patterns in and seasonal drivers of grazing indicate different thermal niche

Quantifying patterns in key ecological processes through space and time is critical to understanding how ecosystems are responding to climate change. Here, we quantified grazing rates of two ecologically important sea urchin species at five locations across their latitudinal range, as well as seasonally throughout the year at a single high-latitude location. The two sea urchins showed subtle, yet contrasting patterns in grazing rates across the latitudinal and thermal gradient. We discuss these findings with respect to differences in the species life history traits, thermal niche, and potential responses to ocean warming.

Grazing rates of sea urchins throughout the study were characterised by considerable variability in rates ranging from 0 to complete consumption of algal assays within the exposure period - consistent with previous studies that have examined patterns of herbivory across broad spatio-temporal gradients^48,49^. Despite this variability, grazing rates of *Centrostephanus rodgersii*showed a peak in the centre of its range, across a steep (12°) latitudinal and (8°C) temperature gradient. This pattern is consistent with the characteristic unimodal shape of thermal performance curve (TPC^9^;), and suggests grazing rates are responsive to differences in temperature. Unlike conventional TPC’s performed on a single organism or population, this pattern was recorded across different populations and suggests the influence of temperature on grazing rates may be conserved among populations for *C. rodgersii*[e.g^19,60^.. The hypothesis that *C. rodgersii* demonstrate a conserved thermal niche is consistent with the species’ highly dispersive reproductive mode and high genetic connectivity among populations, both of which are properties that are commonly associated with niche conservatism^61,62^. Thermal tolerance of adult *C. rodgersii* has not previously been investigated, however thermal limits of *C. rodgersii* larval development have been studied in different populations across its native and extended range. Interestingly larval thermal tolerance across latitude shows different patterns to what we observed. The temperature range for successful larval development decreases from warmer to cooler latitudes^53,63–65^, and suggests thermal plasticity in larval development. How well larval thermal tolerances of *C. rodgersii* reflect those of adult life stages is not known.

A conserved thermal niche for *C. rodgersii* is also supported by the parallel peaks in both grazing rates and abundance, which suggests that this temperature range may represent the species’ thermal optimum. In optimal physiological conditions, organisms may be able to allocate more resources to functions such as reproduction and survival, resulting in increased abundances. Certainly the up-regulation of physiological protective mechanisms in response to thermal stress (cool or warm) often involves high energy demands and can simultaneously cause direct costs to the organism, such as reduced reproductive output, non-adaptive metabolic depression and mortality^66,67^. Increased urchin abundances in this region may also be due to local or regional factors that decrease mortality or increase larval supply and settlement, which may include low predator abundances^68,69^ or local and regional current regimes that work to increase larval supply and settlement (Cresswell et al., In prep.). Ultimately, the higher abundances in this area may result from a combination of all these factors.

A conserved thermal niche further aligns with the output from seasonal models, where temperature was a key driver of grazing rates for *C. rodgersii*. Temperature had a significant and positive effect on grazing rates within the range of observed seasonal temperatures (between 12.5 to 18.0 °C). The positive effect of temperature on grazing rates is likely a result of the relationship between temperature and metabolic rates. As temperature increases, so do metabolic rates of ectothermic species, which increases their energy demand^8^. With the assumption that the positive effect of temperature on grazing rates across seasonal temperature cycles also holds across spatial temperature gradients, increasing temperatures likely contributed to the increased grazing rates we observed between cool and centre-range populations. Increased metabolic rates cannot, however, explain the downturn in grazing rates in warm-edge locations. Other studies have found reduced grazing under increased thermal stress^70^, which may result from the suppression of some physiological functions in order to allocate reserves to stress-protection mechanisms^71^.Controlled laboratory experiments, and observations of seasonal grazing rates at different locations (particularly where temperatures reach higher than those observed at site E) would provide further insight into the mechanisms underpinning reduced rates in warm-edge locations.

Although temperature was important in all of the models, other factors cannot be entirely discounted. Models including the nutritional quality of kelp (i.e.. nitrogen*gonad index and nitrogen*temperature), displayed similar AIC scores, suggesting these factors are likely important as well. Further work under controlled laboratory experiments would help isolate how these mechanisms interact with temperature to affect urchin grazing responses.

In contrast to *C. rodgersii*, *H. erythrogramma* displayed constant grazing rates across latitude. A lack of variation in performance between populations experiencing different thermal conditions implies that individuals have adjusted their physiological requirements to local conditions^20^, and we thus hypothesise that *H. erythrogramma* demonstrate a locally acclimated or adapted thermal niche. This hypothesis is consistent with the species short larval phase (i.e. limited dispersal) and presence of genetically distinct populations across its range^72^. Adjustment of grazing rates to local temperature conditions also aligns with other comparative studies of *H. erythrogramma* thermal performance, albeit from embryos, where it was observed warm-edge populations displayed significantly higher tolerance than those at higher latitudes^73^. This and all other studies investigating thermal limits of *H. erythrogramma*have taken place within the species warm range edge [e.g^66,74,75^., and while no comparative work has previously been completed on cool-edge populations we might expect these populations to show even lower tolerance than their warm-edge counterparts.

The hypothesis that *H. erythrogramma* have acclimated or adapted to local temperature conditions is also supported by the non-significant effect of temperature demonstrated in seasonal models. However, the lack of trend in grazing across latitude is interesting in context of the significant interaction between gonad index and nitrogen content (i.e. nutritional vaule) of *E. radiata* across seasons. Relative to seasonal data, *H. erythrogramma* in the latitudinal analysis had consistently low gonad indices, and nitrogen content of *E. radiata* was similarly low across all sites. The consistency of these values between sites may contribute to the lack of trend in grazing rates across latitude, particularly when considering that temperature was not an important driver. Notably, the nitrogen values and C:N ratios we recorded indicate that *E. radiata* is highly nitrogen limited across its range in southeast Australia during summer. Nitrogen-rich kelp will typically contain 2-3 fold more nitrogen than recorded in our samples, and a C:N ratio < 20^76^. Whether larger differences in nitrogen content of *E. radiata* across latitude may occur at different times of the year, and how this may influence the pattern in grazing across latitude remains unknown.

### Ecological implications in a warmer future

In the context of continued ocean warming, a conserved thermal niche, such as that displayed by *C. rodgersii*, suggests that warm-edge populations may be more susceptible to warming than those in the central and cool range-edge. That is, warming ocean temperature may approach the upper thermal limits of warm-edge populations, leading to reduced performance, while warming conditions in areas further poleward may be favourable for *C. rodgersii *performance. In fact, a cool-edge range expansion is already evident in the recent range-extension of this species^27^, and, although a warm-edge contraction has not yet been documented^77^, predicts this eventuality over coming decades.

Ecologically, the greatest grazing pressure of *C. rodgersii* occurs within the central range sites, resulting from the compounding effects of highest grazing rates and highest abundances. This region of highest observed grazing impact aligns with work by^35^ which found an increase in barrens extent across their study region from warm- to mid-latitudes. Considering a conserved thermal niche, our findings indicate that with continued warming the region of highest grazing impact, currently between -32°and -38°, will shift progressively pole-ward, tracking the pace of ocean warming. Such a shift in the distribution of grazing pressure would place increasing stress on remaining kelp ecosystems, which are already under threat from warming ocean temperatures and more frequent heatwave events (Shi et al. In press^78^).

For *H. erythrogramma*, populations at the cool- and warm-edge of their range are expected to demonstrate similar susceptibility to warming. For a given temperature anomaly, declines may be observed in this species across its entire range. However, depending on local conditions, the absolute upper and lower thermal limits may vary throughout the species range, resulting in differing fitness and survival when cool and warm-edge populations are exposed to the same absolute temperature conditions^20,79^. This variation may provide opportunity for some populations to adjust to warmer conditions, although such acclimatisation will depend on multiple other factors, including the rate of thermal change and whether variation in thermal limits is phenotypically or genetically based^80^.

For both *C. rodgersii* and *H. erythrograma* our data collected across latitude and season provide the first broad-scale field measurements of feeding rates for these important species. They have allowed us to identify patterns and generate hypotheses about each species thermal niche and their potential response to continued ocean warming. These hypotheses are supported by our current understanding of these species biology and ecology, however further experiential work (e.g. common garden and controlled laboratory experiments) is required to effectively isolate the impact of temperature on the grazing rates of these species and to better understand how it changes across their geographic range.

## Conclusions

Quantifying feeding rates of two barrens-forming urchins across latitudinal and seasonal gradients has revealed patterns that reflect a contrasting thermal niche between species. These results provide information that is critical for conservation and management of these species, as well as their ecological impact on temperate reef ecosystems. Our results suggest that grazing impacts of *C. rodgersii *will increase at the cool-edge of this species range, with compounding effects of both increased abundance and increased individual grazing rates as warming continues. At this cool range-edge, mitigation strategies will need to be considered to counter the additional pressure this will put on remaining kelp ecosystems. Conversely impacts from *H. erythrogramma* are likely to remain more constant, as populations across its range are equally vulnerable to warming. For this species, management efforts may focus on identifying more thermally tolerant populations for conservation purposes. In a broader context, this work highlights the importance of identifying patterns in key ecological processes across broad spatial and temporal scales, as this information is invaluable in predicting and managing climate change impacts in marine ecosystems.

## Supplementary Information

Supplementary Information.

## Data Availability

The dataset created for this paper is accessible at https://github.com/cbutler2/grazing-latitude-season. Reef Life Survey data is accessed at the Australian Ocean Data Network portal https://portal.aodn.org.au/ (accessed on 17/01/2023). NOAA OISST data can be accessed at doi:10.7289/V5SQ8XB5.

## References

[1] Lenoir, J. et al. Species better track climate warming in the oceans than on land. *Nature Ecology Evolution* **4**, 1044–1059 (2020) https://www.nature.com/articles/s41559-020-1198-2. 32451428 10.1038/s41559-020-1198-2

[2] Poloczanska, E. S. et al. Global imprint of climate change on marine life. *Nat. Clim. Chang.* **3**, 919–925 (2013) http://www.nature.com/articles/nclimate1958.

[3] Pecl, G. T. et al. Biodiversity redistribution under climate change: Impacts on ecosystems and human well-being. *Science* **355**, eaai9214 (2017) https://www.sciencemag.org/lookup/doi/10.1126/science.aai9214. 28360268 10.1126/science.aai9214

[4] Poore, A. G. B. et al. Global patterns in the impact of marine herbivores on benthic primary producers. *Ecology Lett.* **15**, 912–922 (2012). 10.1111/j.1461-0248.2012.01804.x 22639820

[5] Frank, D. A., McNaughton, S. J. & Tracy, B. F. The Ecology of the Earth’s Grazing Ecosystems. *Bioscience* **48**, 513–521 (1998) https://academic.oup.com/bioscience/article-lookup/doi/10.2307/1313313.

[6] Burkepile, D. E. Comparing aquatic and terrestrial grazing ecosystems: is the grass really greener?. *Oikos* **122**, 306–312 (2013) https://onlinelibrary.wiley.com/doi/10.1111/j.1600-0706.2012.20716.x.

[7] Dublin, H. T., Sinclair, A. & McGlade, J. Elephants and Fire as Causes of Multiple Stable States in the Serengeti-Mara Woodlands. *J. Anim. Ecol.* **59**, 1147 (1990) https://www.jstor.org/stable/5037?origin=crossref.

[8] Gillooly, J. F. Effects of Size and Temperature on Metabolic Rate. *Science* **293**, 2248–2251 (2001) https://www.sciencemag.org/lookup/doi/10.1126/science.1061967. 11567137 10.1126/science.1061967

[9] Angilletta, M. J., Niewiarowski, P. H. & Navas, C. A. The evolution of thermal physiology in ectotherms. *J. Therm. Biol* **27**, 249–268 (2002) https://linkinghub.elsevier.com/retrieve/pii/S0306456501000948.

[10] Pagès, J. F. et al. Contrasting effects of ocean warming on different components of plant-herbivore interactions. *Mar. Pollut. Bull.* **134**, 55–65 (2018) https://linkinghub.elsevier.com/retrieve/pii/S0025326X17308822. 29074253 10.1016/j.marpolbul.2017.10.036

[11] O’Connor, M. I. Warming strengthens an herbivore-plant interaction. *Ecology* **90**, 388–398 (2009) https://esajournals.onlinelibrary.wiley.com/doi/10.1890/08-0034.1. 19323223 10.1890/08-0034.1

[12] Tylianakis, J. M., Didham, R. K., Bascompte, J. & Wardle, D. A. Global change and species interactions in terrestrial ecosystems. *Ecol. Lett.* **11**, 1351–1363 (2008) https://onlinelibrary.wiley.com/doi/10.1111/j.1461-0248.2008.01250.x. 19062363 10.1111/j.1461-0248.2008.01250.x

[13] Birkemoe, T., Bergmann, S., Hasle, T. E. & Klanderud, K. Experimental warming increases herbivory by leaf-chewing insects in an alpine plant community. *Ecol. Evol.* **6**, 6955–6962 (2016) https://onlinelibrary.wiley.com/doi/10.1002/ece3.2398. 28725372 10.1002/ece3.2398 PMC5513215

[14] Rezende, E. L. & Bozinovic, F. Thermal performance across levels of biological organization. *Philosophical Transa. Royal Soc. B: Biol. Sci.* **374**, 10. 10.1098/rstb.2018.0549 (2019). 10.1098/rstb.2018.0549 PMC6606466 31203764

[15] Boada, J. et al. Immanent conditions determine imminent collapses: nutrient regimes define the resilience of macroalgal communities. *Proceed. Royal Soc. B: Biol. Sci.* **284**, 20162814 (2017) https://royalsocietypublishing.org/doi/10.1098/rspb.2016.2814. 10.1098/rspb.2016.2814 PMC5378086 28330920

[16] Berner, D., Blanckenhorn, W. U. & Körner, C. Grasshoppers cope with low host plant quality by compensatory feeding and food selection: N limitation challenged. *Oikos* **111**, 525–533 (2005) https://onlinelibrary.wiley.com/doi/10.1111/j.1600-0706.2005.14144.x.

[17] Lindmark, M., Ohlberger, J. & Gårdmark, A. Optimum growth temperature declines with body size within fish species. *Glob. Change Biol.* **28**, 2259–2271 (2022). 10.1111/gcb.16067 35060649

[18] Walker, C. W., Lesser, M. P. & Unuma, T. Gametogenesis in regular sea urchins: Structural, functional, and molecular/genomic biology. In *Developments in Aquaculture and Fisheries Science*, vol. 43, 29–50 (Elsevier, 2020). https://linkinghub.elsevier.com/retrieve/pii/B9780128195703000032.

[19] Dunphy, B. J., Ragg, N. L. C. & Collings, M. G. Latitudinal comparison of thermotolerance and HSP70 production in F2 larvae of the Greenshell mussel ( *Perna canaliculus* ). *Journal of Experimental Biology* jeb.076729 (2012). https://journals.biologists.com/jeb/article/doi/10.1242/jeb.076729/257929/Latitudinal-comparison-of-thermotolerance-and. 10.1242/jeb.076729 23239885

[20] Bennett, S., Wernberg, T., Arackal Joy, B., de Bettignies, T. & Campbell, A. H. Central and rear-edge populations can be equally vulnerable to warming. *Nat. Commun.* **6**, 10280 (2015) http://www.nature.com/articles/ncomms10280. 26691184 10.1038/ncomms10280 PMC4703895

[21] Duarte, C. M. et al. Global estimates of the extent and production of macroalgal forests. *Glob. Ecol. Biogeogr.* **31**, 1422–1439 (2022) https://onlinelibrary.wiley.com/doi/abs/10.1111/geb.13515.

[22] Pessarrodona, A. et al. Global seaweed productivity. *Science Advances* (2022). 10.1126/sciadv.abn2465 PMC9473579 36103524

[23] Teagle, H., Hawkins, S. J., Moore, P. J. & Smale, D. A. The role of kelp species as biogenic habitat formers in coastal marine ecosystems. *J. Exp. Mar. Biol. Ecol.* **492**, 81–98 (2017) https://www.sciencedirect.com/science/article/pii/S0022098117300540.

[24] Wernberg, T. & Filbee-Dexter, K. Missing the marine forest for the trees. *Mar. Ecol. Prog. Ser.* **612**, 209–215 (2019) https://www.int-res.com/abstracts/meps/v612/meps12867.

[25] Ling, S. D. et al. Global regime shift dynamics of catastrophic sea urchin overgrazing. *Philosophical Transa. Royal Soc. B: Biol. Sci.* **370**, 20130269 (2015) https://royalsocietypublishing.org/doi/10.1098/rstb.2013.0269.

[26] Vergés, A. et al. The tropicalization of temperate marine ecosystems: climate-mediated changes in herbivory and community phase shifts. *Proceed. Royal Soc. B: Biol. Sci.* **281**, 20140846 (2014) https://royalsocietypublishing.org/doi/10.1098/rspb.2014.0846. 10.1098/rspb.2014.0846 PMC4100510 25009065

[27] Ling, S. D. & Keane, J. P. Climate-driven invasion and incipient warnings of kelp ecosystem collapse. *Nat. Commun.* **15**, 400 (2024) https://www.nature.com/articles/s41467-023-44543-x. 38195631 10.1038/s41467-023-44543-x PMC10776680

[28] Ling, S., Ibbott, S. & Sanderson, J. Recovery of canopy-forming macroalgae following removal of the enigmatic grazing sea urchin Heliocidaris erythrogramma. *J. Exp. Mar. Biol. Ecol.* **395**, 135–146 (2010) https://linkinghub.elsevier.com/retrieve/pii/S002209811000359X.

[29] Keesing, J. K. Heliocidaris erythrogramma. In *Sea Urchins: Biology and Ecology*, vol. 43, 537–552 (Elsevier, 2020), fourth edn. https://linkinghub.elsevier.com/retrieve/pii/B9780128195703000305.

[30] Byrne, M. & Andrew, N. L. Centrostephanus rodgersii and Centrostephanus tenuispinus. In *Sea Urchins: Biology and Ecology*, vol. 43, 379–396 (Elsevier, 2020), fourth edn. https://linkinghub.elsevier.com/retrieve/pii/B9780128195703000226.

[31] Huggett, M. J., King, C. K., Williamson, J. E. & Steinberg, P. D. Larval development and metamorphosis of the Australian diadematid sea urchin Centrostephanus rodgersii. *Invertebrate Reproduction & Development* **47**, 197–204 (2005) http://www.tandfonline.com/doi/abs/10.1080/07924259.2005.9652160.

[32] Mos, B., Byrne, M. & Dworjanyn, S. A. Effects of low and high pH on sea urchin settlement, implications for the use of alkali to counter the impacts of acidification. *Aquaculture* **528**, 735618 (2020) https://www.sciencedirect.com/science/article/pii/S0044848620310103.

[33] Williams, D. & Anderson, D. The reproductive system, embryonic development, larval development and metamorphosis of the sea urchin Heliocidaris erythrogramma (Val.) (Echinoidea : Echinometridae). *Australian Journal of Zoology* **23**, 371 (1975). http://www.publish.csiro.au/?paper=ZO9750371.

[34] Kriegisch, N., Reeves, S. E., Flukes, E. B., Johnson, C. R. & Ling, S. D. Drift-kelp suppresses foraging movement of overgrazing sea urchins. *Oecologia* **190**, 665–677 (2019) http://link.springer.com/10.1007/s00442-019-04445-6. 31250188 10.1007/s00442-019-04445-6

[35] Glasby, T. M. & Gibson, P. T. Decadal dynamics of subtidal barrens habitat. *Mar. Environ. Res.* **154**, 104869 (2020) https://linkinghub.elsevier.com/retrieve/pii/S0141113619306026. 31928986 10.1016/j.marenvres.2019.104869

[36] Ridgway, K. R. Long-term trend and decadal variability of the southward penetration of the East Australian Current. *Geophysical Research Letters* **34**, n/a–n/a (2007). http://doi.wiley.com/10.1029/2007GL030393.

[37] Ridgway, K. & Dunn, J. Mesoscale structure of the mean East Australian Current System and its relationship with topography. *Prog. Oceanogr.* **56**, 189–222 (2003) https://linkinghub.elsevier.com/retrieve/pii/S0079661103000041.

[38] R Core Team. *R: A Language and Environment for Statistical Computing*. R Foundation for Statistical Computing, Vienna, Austria (2024). https://www.R-project.org/.

[39] Vanderklift, M. & Wernberg, T. Stable isotopes reveal a consistent consumer-diet relationship across hundreds of kilometres. *Mar. Ecol. Prog. Ser.* **403**, 53–61 (2010) http://www.int-res.com/abstracts/meps/v403/p53-61/.

[40] Caley, A., Vergés, A., Byrne, M. & Marzinelli, E. *Urchins roe quality, morphology and diet vary with depth and distance from kelp*. Ph.D. thesis, University of New South Wales, Sydney (2021).

[41] Vanderklift, M. & Kendrick, G. Contrasting influence of sea urchins on attached and drift macroalgae. *Mar. Ecol. Prog. Ser.* **299**, 101–110 (2005) http://www.int-res.com/abstracts/meps/v299/p101-110/.

[42] Cooper, A. T. & Oh, E. S. NRMN Database QA/QC Protocols (2023). https://catalogue-imos.aodn.org.au/geonetwork/srv/api/records/bc8987aa-43ac-46a9-b5cf-de0cd4c097b8.

[43] Edgar, G. J. et al. Establishing the ecological basis for conservation of shallow marine life using Reef Life Survey. *Biol. Cons.* **252**, 108855 (2020) https://linkinghub.elsevier.com/retrieve/pii/S0006320720309137.

[44] Britton, D. et al. Seasonal and site-specific variation in the nutritional quality of temperate seaweed assemblages: implications for grazing invertebrates and the commercial exploitation of seaweeds. *J. Appl. Phycol.* **33**, 603–616 (2021) https://link.springer.com/10.1007/s10811-020-02302-1.

[45] Wood, S. *Generalized additive models: An introduction with R* (Chapman and Hall, Boca Raton, Florida, 2017), 2nd edn. 10.1201/9781315370279.

[46] Rigby, R. A. & Stasinopoulos, D. M. Generalized Additive Models for Location, Scale and Shape. *J. R. Stat. Soc.: Ser. C: Appl. Stat.* **54**, 507–554 (2005) https://academic.oup.com/jrsssc/article/54/3/507/7113027.

[47] Wood, S. N., Pya, N. & Säfken, B. Smoothing Parameter and Model Selection for General Smooth Models. *J. Am. Stat. Assoc.* **111**, 1548–1563 (2016) https://www.tandfonline.com/doi/full/10.1080/01621459.2016.1180986.

[48] Bennett, S. & Bellwood, D. R. Latitudinal variation in macroalgal consumption by fishes on the Great Barrier Reef. *Mar. Ecol. Prog. Ser.* **426**, 241–252 (2011) https://www.int-res.com/abstracts/meps/v426/meps09016.

[49] Santana-Garcon, J. et al. Tropicalization shifts herbivore pressure from seagrass to rocky reef communities. *Proceed. Royal Soc. B: Biol. Sci.* **290**, 20221744 (2023) https://royalsocietypublishing.org/doi/10.1098/rspb.2022.1744. 10.1098/rspb.2022.1744 PMC9832549 36629100

[50] Ling, S. D., Johnson, C. R., Ridgway, K., Hobday, A. J. & Haddon, M. Climate-driven range extension of a sea urchin: inferring future trends by analysis of recent population dynamics. *Glob. Change Biol.* **15**, 719–731 (2009) http://doi.wiley.com/10.1111/j.1365-2486.2008.01734.x.

[51] Cresswell, K. A. et al. When overfishing is the sustainable option. *Nature Sustainability* 1–10 (2025). https://www.nature.com/articles/s41893-025-01526-8. Publisher: Nature Publishing Group.

[52] Schuster, J. M., Kurt Gamperl, A., Gagnon, P. & Bates, A. E. Distinct realized physiologies in green sea urchin ( Strongylocentrotus droebachiensis ) populations from barren and kelp habitats. *FACETS* **7**, 822–842 (2022).

[53] Ling, S. D., Johnson, C. R., Frusher, S. & King, C. K. Reproductive potential of a marine ecosystem engineer at the edge of a newly expanded range. *Glob. Change Biol.* **14**, 907–915 (2008) http://doi.wiley.com/10.1111/j.1365-2486.2008.01543.x.

[54] Hijmans, R. J. *geosphere: Spherical Trigonometry* (2024). https://CRAN.R-project.org/package=geosphere. R package version 1.5-20.

[55] Nunes, L. T. et al. Predicting the effects of body size, temperature and diet on animal feeding rates. *Functional Ecology* **1365–2435**, 13872 (2021).

[56] Shokri, M. et al. Metabolic rate and climate change across latitudes: evidence of mass-dependent responses in aquatic amphipods. *J. Exp. Biol.* **225**, jeb244842 (2022). 36337048 10.1242/jeb.244842 PMC9720750

[57] Tredennick, A. T., Hooker, G., Ellner, S. P. & Adler, P. B. A practical guide to selecting models for exploration, inference, and prediction in ecology. Ecology 102, e03336 (2021). doi.org/10.1002/ecy.3336. 10.1002/ecy.3336 PMC8187274 33710619

[58] Venables, W. & Ripley, B. *Modern Applied Statistics with S* (Springer, New York, 2002), fourth edition edn. https://www.stats.ox.ac.uk/pub/MASS4/.

[59] Davison, A. & Hinkley, D. V. *Bootstrap methods and their application*. Cambridge Series in Statistical and Probabalistic Mathematics (Cambridge University Press, 1997).

[60] Beca-Carretero, P., Olesen, B., Marbà, N. & Krause-Jensen, D. Response to experimental warming in northern eelgrass populations: comparison across a range of temperature adaptations. *Mar. Ecol. Prog. Ser.* **589**, 59–72 (2018) http://www.int-res.com/abstracts/meps/v589/p59-72/.

[61] Bennett, S., Duarte, C. M., Marba, N. & Wernberg, T. Integrating within-species variation in thermal physiology into climate change ecology. *Philosophical Transa. Royal Soc. B: Biol. Sci.* **347**, 10 (2019). 10.1098/rstb.2018.0550 PMC6606463 31203756

[62] Parsons, K. E. *The Role of dispersal ability in the phenotypic differentiation and plasticity of two marine gastropods II* (Growth. J. Exp. Mar. Biol, 1998). 10.1007/s004420050181 28307236

[63] Pecorino, D., Lamare, M. D., Barker, M. F. & Byrne, M. How does embryonic and larval thermal tolerance contribute to the distribution of the sea urchin Centrostephanus rodgersii (Diadematidae) in New Zealand?. *J. Exp. Mar. Biol. Ecol.* **445**, 120–128 (2013) https://linkinghub.elsevier.com/retrieve/pii/S0022098113001676.

[64] Byrne, M., Gall, M. L., Campbell, H., Lamare, M. D. & Holmes, S. P. Staying in place and moving in space: Contrasting larval thermal sensitivity explains distributional changes of sympatric sea urchin species to habitat warming. *Global Change Biology* gcb.16116 (2022). https://onlinelibrary.wiley.com/doi/10.1111/gcb.16116. 10.1111/gcb.16116 35108424

[65] Hardy, N. A. et al. Thermal tolerance of early development in tropical and temperate sea urchins: inferences for the tropicalization of eastern Australia. *Mar. Biol.* **161**, 395–409 (2014) http://link.springer.com/10.1007/s00227-013-2344-z.

[66] Minuti, J. J., Byrne, M., Hemraj, D. A. & Russell, B. D. Capacity of an ecologically key urchin to recover from extreme events: Physiological impacts of heatwaves and the road to recovery. *Sci. Total Environ.* **785**, 147281 (2021) https://linkinghub.elsevier.com/retrieve/pii/S0048969721023524. 33933766 10.1016/j.scitotenv.2021.147281

[67] Pörtner, H. et al. Trade-Offs in Thermal Adaptation: The Need for a Molecular to Ecological Integration. *Physiol. Biochem. Zool.* **79**, 295–313 (2006) http://www.journals.uchicago.edu/doi/10.1086/499986. 16555189 10.1086/499986

[68] Filbee-Dexter, K. & Scheibling, R. Sea urchin barrens as alternative stable states of collapsed kelp ecosystems. *Mar. Ecol. Prog. Ser.* **495**, 1–25 (2014) http://www.int-res.com/abstracts/meps/v495/p1-25/.

[69] Wharton, W. G. & Mann, K. H. Relationship Between Destructive Grazing by the Sea Urchin, Strongylocentrotus droebachiensis, and the Abundance of American Lobster, Homarus americanus, on the Atlantic Coast of Nova Scotia. *Can. J. Fish. Aquat. Sci.* **38**, 1339–1349 (1981) http://www.nrcresearchpress.com/doi/10.1139/f81-180.

[70] Hill, S. K. & Lawrence, J. M. Interactive effects of temperature and nutritional condition on the energy budgets of the sea urchins Arbacia punctulata and Lytechinus variegatus (Echinodermata: Echinoidea). *J. Marine Biol. Association UK* **86**, 783–790 (2006).

[71] Pan, T.-C.F., Applebaum, S. L. & Manahan, D. T. Experimental ocean acidification alters the allocation of metabolic energy. *Proceed. National Academy Sci.* **112**, 4696–4701 (2015). 10.1073/pnas.1416967112 PMC4403215 25825763

[72] McMillan, W. O., Raff, R. A. & Palumbi, S. R. Population genetic consequences of developmental evolution in sea urchins (genus Heliocidaris). *Evolution* **46**, 1299–1312 (1992) https://onlinelibrary.wiley.com/doi/10.1111/j.1558-5646.1992.tb01125.x. 28568989 10.1111/j.1558-5646.1992.tb01125.x

[73] Byrne, M., Selvakumaraswamy, P., Ho, M., Woolsey, E. & Nguyen, H. Sea urchin development in a global change hotspot, potential for southerly migration of thermotolerant propagules. *Deep Sea Res. Part II* **58**, 712–719 (2011) https://linkinghub.elsevier.com/retrieve/pii/S0967064510002171.

[74] Harianto, J., Nguyen, H. D., Holmes, S. P. & Byrne, M. The effect of warming on mortality, metabolic rate, heat-shock protein response and gonad growth in thermally acclimated sea urchins (Heliocidaris erythrogramma). *Mar. Biol.* **165**, 96 (2018) http://link.springer.com/10.1007/s00227-018-3353-8.

[75] Harianto, J., Aldridge, J., Torres Gabarda, S. A., Grainger, R. J. & Byrne, M. Impacts of Acclimation in Warm-Low pH Conditions on the Physiology of the Sea Urchin Heliocidaris erythrogramma and Carryover Effects for Juvenile Offspring. *Front. Mar. Sci.* **7**, 588938 (2021) https://www.frontiersin.org/articles/10.3389/fmars.2020.588938/full.

[76] Hurd, C. L., Harrison, P. J., Bischof, K. & Lobban, C. S. Nutrients. In *Seaweed Ecology and Physiology* (Cambridge University Press, Cambridge, 2014), 2 edn. http://ebooks.cambridge.org/ref/id/CBO9781139192637.

[77] Davis, T. R., Knott, N. A., Champion, C. & Przeslawski, R. Impacts of Climate Change on Densities of the Urchin Centrostephanus rodgersii Vary among Marine Regions in Eastern Australia. *Diversity* **15**, 419 (2023) https://www.mdpi.com/1424-2818/15/3/419.

[78] Shi, J. et al. Warm edge kelp populations show elevated volatility to marine heatwaves. Ecology Letters (in press). 10.1111/ele.70307 PMC12828870 41574748

[79] Sanford, E. & Kelly, M. W. Local Adaptation in Marine Invertebrates. *Ann. Rev. Mar. Sci.* **3**, 509–535 (2011) https://www.annualreviews.org/doi/10.1146/annurev-marine-120709-142756. 21329215 10.1146/annurev-marine-120709-142756

[80] Donelson, J. M., Munday, P. L., McCormick, M. I. & Pitcher, C. R. Rapid transgenerational acclimation of a tropical reef fish to climate change. *Nat. Clim. Chang.* **2**, 30–32 (2012) https://www.nature.com/articles/nclimate1323.

