# Supplementary material for: Contrasting patterns in kelp consumption across latitude by two barren forming sea urchin species
Source: Sci Rep. 2026 Feb 14;16:9069. doi: 10.1038/s41598-025-33714-z (PMC12992891; doi:10.1038/s41598-025-33714-z)
Supplement: Supplementary file 1 — Supplementary Information. [file 41598_2025_33714_MOESM1_ESM.pdf]

9 Supporting information

Contrasting patterns in kelp consumption across latitude by two barren forming sea urchin species.

Claire Butler, Yanheng Wang, Christopher J. Brown, Adriana Vergés, Scott Ling, Catriona L. Hurd, Scott Bennett.

Table S1: Habitat descriptions of each site where urchins were collected. At all sites urchin were collected subtidally in < 10 m depths.

Site	Habitat description
Sawtell (site A)	High profile reef dominated with medium density *E. radiata*, coralline algae and invertebrates.
Forster (site B)	Platform and boulder reef with dense *E. radiata* canopy.
Shellharbour (site C)	Platform and boulder reef with high abundance of *C. rodgersii* in barren patches interspersed with large areas of of dense *E. radiata* and *Phyllospora comosa*. Urchins collected from within macoalgal beds.
Merimbula (site D)	Boulder reef with medium to dense canopy-forming macroalgae (*Ecklonia radiata* , *Cystophora monoliformis*, *Phyllospora comosa*). Canopy-forming habitat interspersed with *Centrostephanus rodgersii* barren patches and mussel beds in deeper (>10 m) depths. Urchins collected from within macoalgal beds.
Fortescue (site E)	Boulder reef with dense macroalgal cover (*E. radiata*, *Phyllospora comosa*, *Cystophora. sp*) over boulder (>1 m) substrate. Some incipient barren patches.

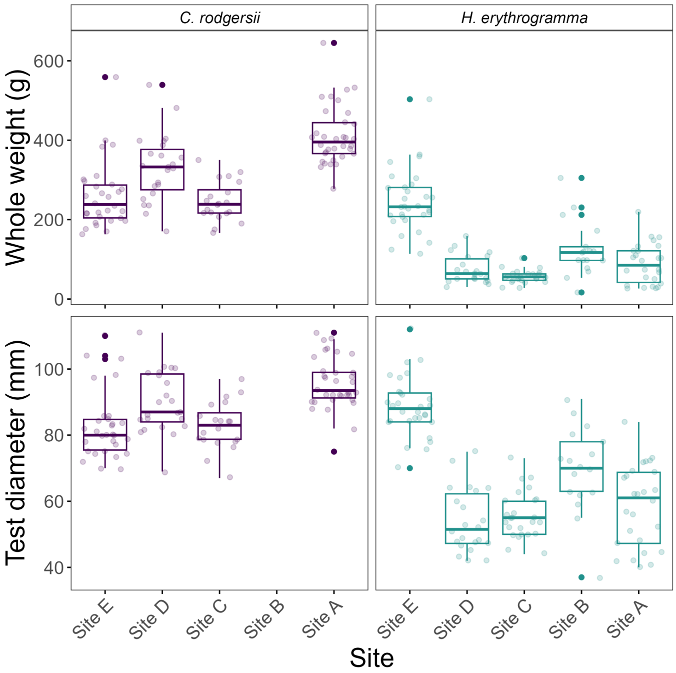

Figure S1: Whole wet weight and test diameter of *C. rodgersii* and *H. erythrogramma* collected at each site across latitude. Points show raw data, while boxplot depicts the median (bold line) and the 25th and 75th percentiles.

Table S2: The mean whole wet weight (g ± SE)of each urchin species used in grazing assays at each location across latitude.

Site	*C. rodgersii*	*H.erythrogramma*
A (Sawtell)	411.4 ± 12.7	89.0 ± 10.0
B (Forster)	-	125.7 ± 17.0
C (Shellharbour)	245.6 ± 11.1	56.6 ± 3.2
D (Merimbula)	330.2 ± 17.8	74.7 ± 7.47
E (Fortescue Bay)	260.1 ± 15.4	246 ± 14.5

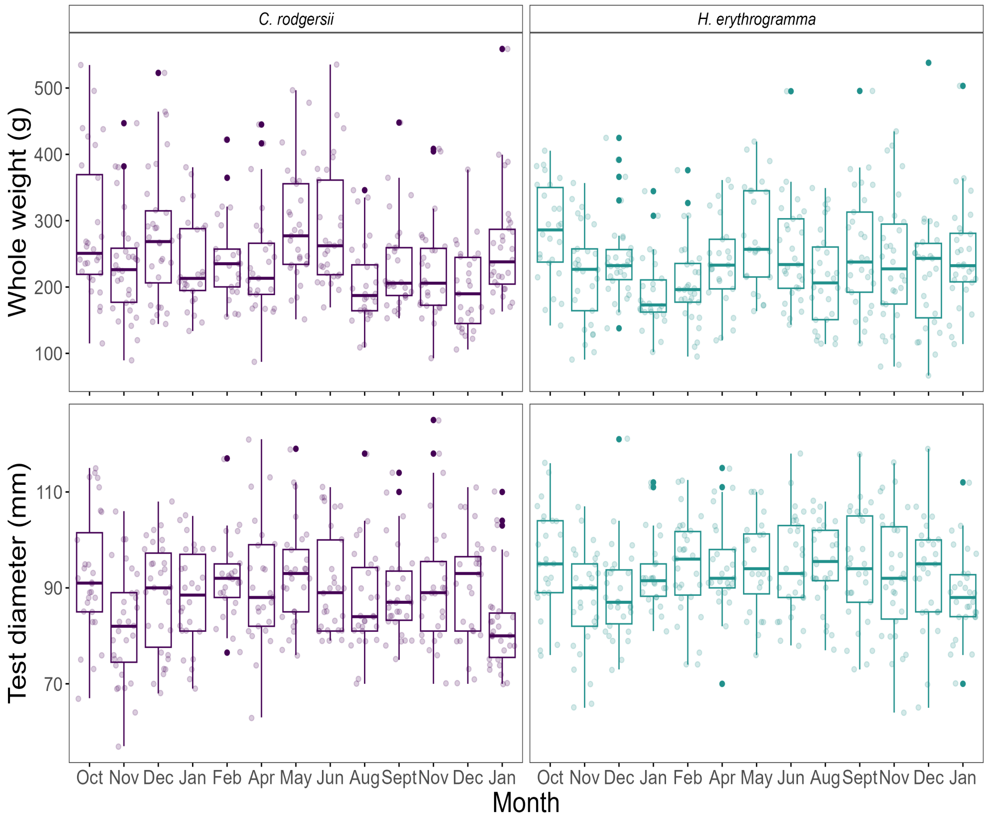

Figure S2: Whole weight and test diameter of *C. rodgersii* and *H. erythrogramma* collected at each sampling period. Points show raw data, while boxplot depicts the median (bold line) and the 25th and 75th percentiles.

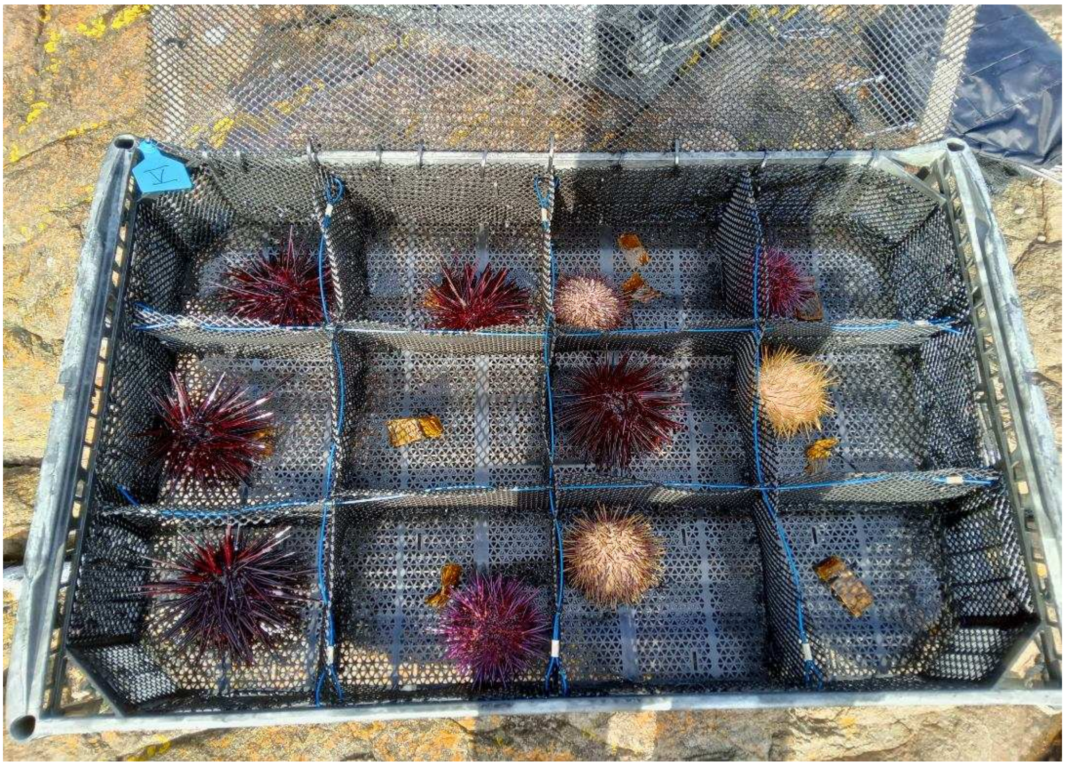

Figure S3: One of the grazing cages used for grazing assays. Urchins were haphazardly placed inside one of 12 individual compartments (265 mm x 235 mm x 110 mm) in one of 6 weighted inclusion cages.Urchins were fed fresh lateral blades of the kelp *E. radiata* that were fastened to the base of each compartment using cable ties. Two compartments contained no urchin to account for natural growth and erosion of kelp over the course of the assay.

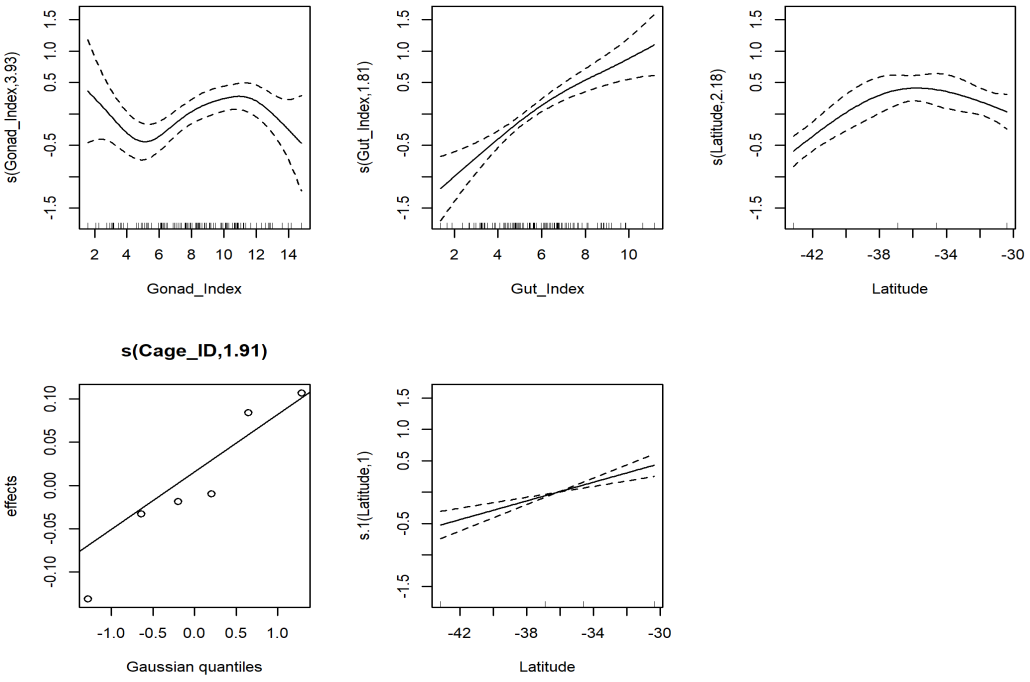

Figure S4: Partial effects plots from the GAM fit to the grazing rates of the sea urchin *C. rodgersii* across latitude using a tweedie distribution. To fit this model, all negative grazing rates were zero-ed. This model shows similar trends and significance of model parameters to the gaussian location-scale model where negative grazing rates were preserved to more accurately represent error estimation.

Table S3: Summary output from the GAM fit to the grazing rates of the sea urchin *C. rodgersii* across latitude using a tweedie distribution. To fit this model, all negative grazing rates were zero-ed. This model shows similar trends and significance of model parameters to the gaussian location-scale model where negative grazing rates were preserved to more accurately represent error estimation.

Component	Term	Estimate	Std Error	t-value	p-value	
A. parametric coefficients	(Intercept)	-0.436	0.094	-4.664	0.0000	***
	(Intercept).1	-0.343	0.072	-4.784	0.0000	***
**Component**	**Term**	**edf**	**Ref. df**	**F-value**	**p-value**	
B. smooth terms	s(Gonad_Index)	3.928	4.839	14.558	0.0133	*
	s(Gut_Index)	1.807	2.262	42.897	0.0000	***
	s(Latitude)	2.176	2.424	38.013	0.0000	***
	s(Cage_ID)	1.911	5.000	3.775	0.0737	.
	s.1(Latitude)	1.000	1.000	23.193	0.0000	***

Signif. codes: 0 <= '***' < 0.001 < '**' < 0.01 < '*' < 0.05

Adjusted R-squared: NA, Deviance explained 0.442

-REML : 137.927, Scale est: 1.000, N: 107

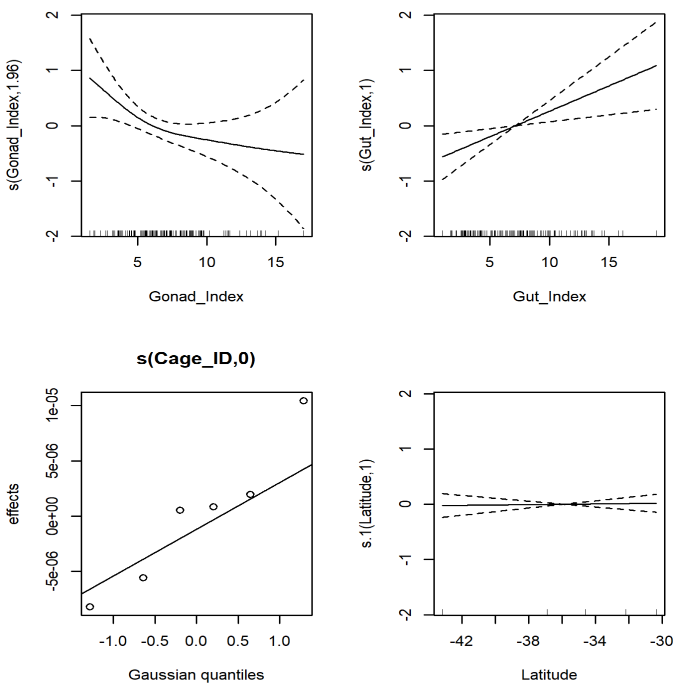

Figure S5: Partial effects plots from the GAM fit to the grazing rates of the sea urchin *H. erythrogramma* across latitude using a tweedie distribution. To fit this model, all negative grazing rates were zero-ed. This model shows similar trends and significance of model parameters to the gaussian location-scale model where negative grazing rates were preserved to more accurately represent error estimation.

Table S4: Summary output from the GAM fit to the grazing rates of the sea urchin *H. erythrogramma* across latitude using a tweedie distribution. To fit this model, all negative grazing rates were zero-ed. This model shows similar trends and significance of model parameters to the gaussian location-scale model where negative grazing rates were preserved to more accurately represent error estimation. Although latitude shows a significant trend, there is no consistent pattern across latitude.

**Component**	**Term**	**Estimate**	**Std Error**	**t-value**	**p-value**	
A. parametric coefficients	(Intercept)	-1.023	0.115	-8.860	0.0000	***
	(Intercept).1	0.226	0.065	3.461	0.0005	***
**Component**	**Term**	**edf**	**Ref. df**	**F-value**	**p-value**	
B. smooth terms	s(Gonad_Index)	1.960	2.482	7.346	0.0613	.
	s(Gut_Index)	1.000	1.000	7.564	0.0060	**
	s(Latitude)	1.000	1.000	0.037	0.8475	
	s(Cage_ID)	0.000	6.000	0.000	0.5509	
	s.1(Latitude)	1.000	1.000	0.046	0.8296	

Signif. codes: 0 <= '***' < 0.001 < '**' < 0.01 < '*' < 0.05

Adjusted R-squared: NA, Deviance explained 0.142

-REML : 208.029, Scale est: 1.000, N: 120

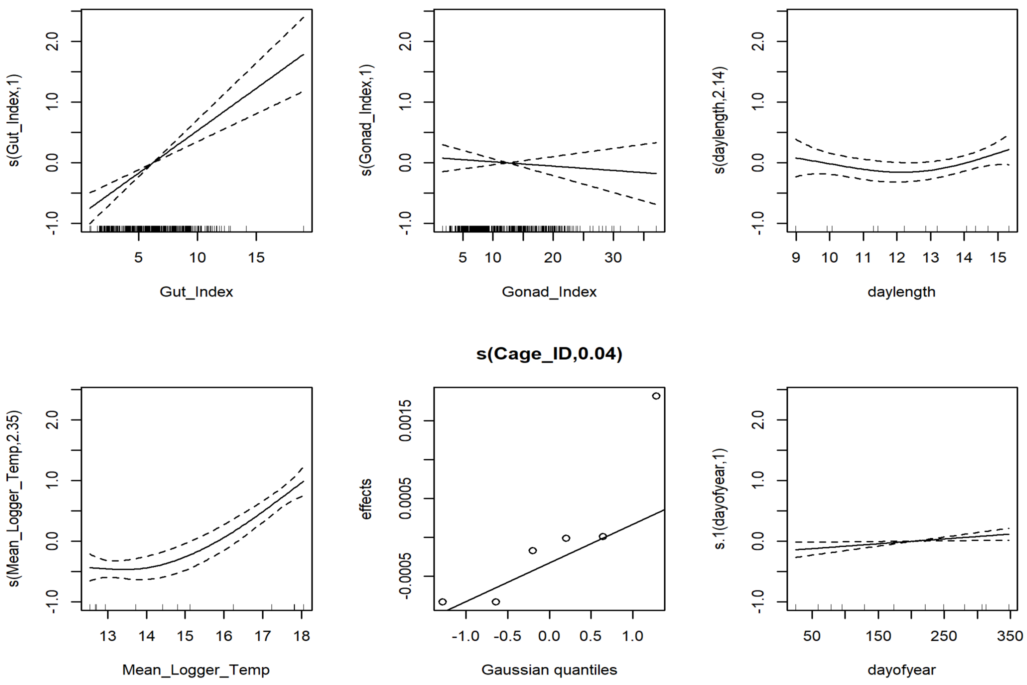

Figure S6: Partial effects plots from the GAM fit to the grazing rates of the sea urchin *C. rodgersii* across seasons using a tweedie distribution. To fit this model, all negative grazing rates were zero-ed. This model shows similar trends and significance of model parameters to the gaussian location-scale model where negative grazing rates were preserved to more accurately represent error estimation.

Table S5: Summary output from the GAM fit to the grazing rates of the sea urchin *C. rodgersii* across season using a tweedie distribution. To fit this model, all negative grazing rates were zero-ed. This model shows similar trends and significance of model parameters to the gaussian location-scale model where negative grazing rates were preserved to more accurately represent error estimation.

Component	Term	Estimate	Std Error	t-value	p-value	
A. parametric coefficients	(Intercept)	0.425	0.478	0.889	0.3739	
	Duration_Exposure	-0.157	0.070	-2.245	0.0248	*
	(Intercept).1	-0.037	0.037	-0.986	0.3240	
**Component**	**Term**	**edf**	**Ref. df**	**F-value**	**p-value**	
B. smooth terms	s(Gut_Index)	1.001	1.001	34.499	0.0000	***
	s(Gonad_Index)	1.000	1.000	0.477	0.4897	
	s(daylength)	2.143	2.643	4.780	0.1851	
	s(Mean_Logger_Temp)	2.348	2.838	105.211	0.0000	***
	s(Cage_ID)	0.038	5.000	0.040	0.3800	
	s.1(dayofyear)	1.000	1.000	5.327	0.0210	*

Signif. codes: 0 <= '***' < 0.001 < '**' < 0.01 < '*' < 0.05

Adjusted R-squared: NA, Deviance explained 0.340

-REML : 528.427, Scale est: 1.000, N: 365

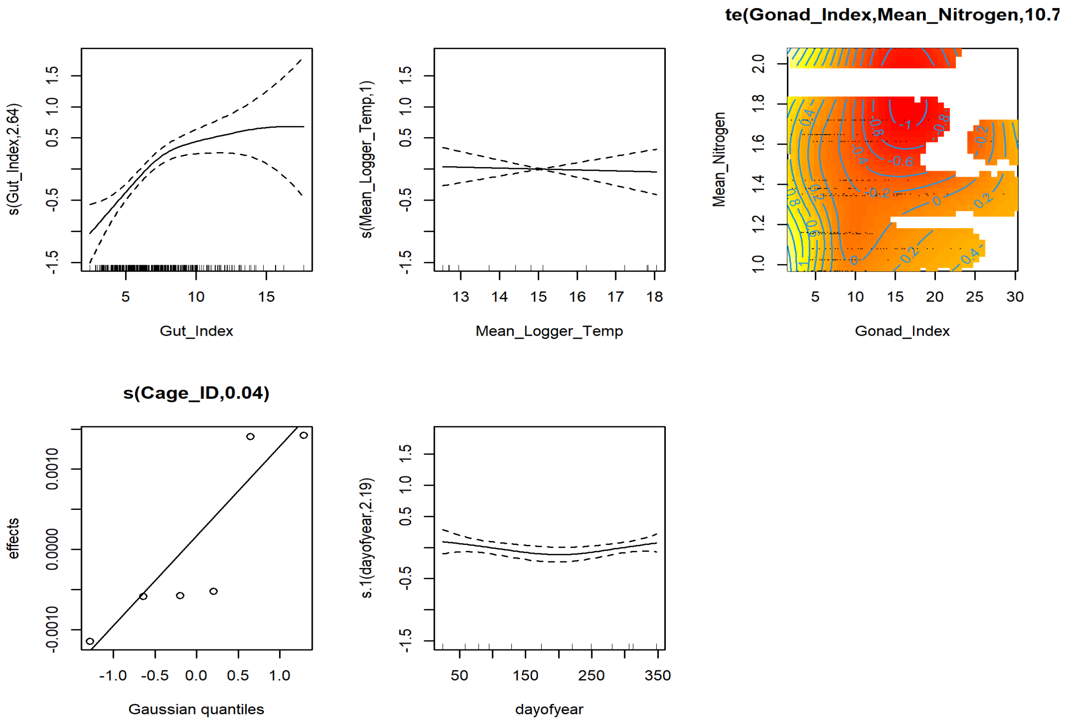

Figure S7: Partial effects plots from the GAM fit to the grazing rates of the sea urchin *H. erythrogramma* across season using a tweedie distribution. To fit this model, all negative grazing rates were zero-ed. This model shows similar trends and significance of model parameters to the gaussian location-scale model where negative grazing rates were preserved to more accurately represent error estimation.

Table S6: Summary output from the GAM fit to the grazing rates of the sea urchin *H. erythrogramma* across season using a tweedie distribution. To fit this model, all negative grazing rates were zero-ed. This model shows similar trends and significance of model parameters to the gaussian location-scale model where negative grazing rates were preserved to more accurately represent error estimation.

Component	Term	Estimate	Std Error	t-value	p-value	
A. parametric coefficients	(Intercept)	0.079	0.487	0.163	0.8707	
	Duration_Exposure	-0.144	0.071	-2.023	0.0431	*
	(Intercept).1	0.115	0.038	3.031	0.0024	**
**Component**	**Term**	**edf**	**Ref. df**	**F-value**	**p-value**	
B. smooth terms	s(Gut_Index)	2.644	3.341	42.413	0.0000	***
	s(Mean_Logger_Temp)	1.001	1.002	0.061	0.8076	
	te(Gonad_Index,Mean_Nitrogen)	10.772	13.486	41.129	0.0001	***
	s(Cage_ID)	0.036	5.000	0.039	0.3467	
	s.1(dayofyear)	2.186	2.731	3.388	0.3076	

Signif. codes: 0 <= '***' < 0.001 < '**' < 0.01 < '*' < 0.05

Adjusted R-squared: NA, Deviance explained 0.250

-REML : 585.838, Scale est: 1.000, N: 363

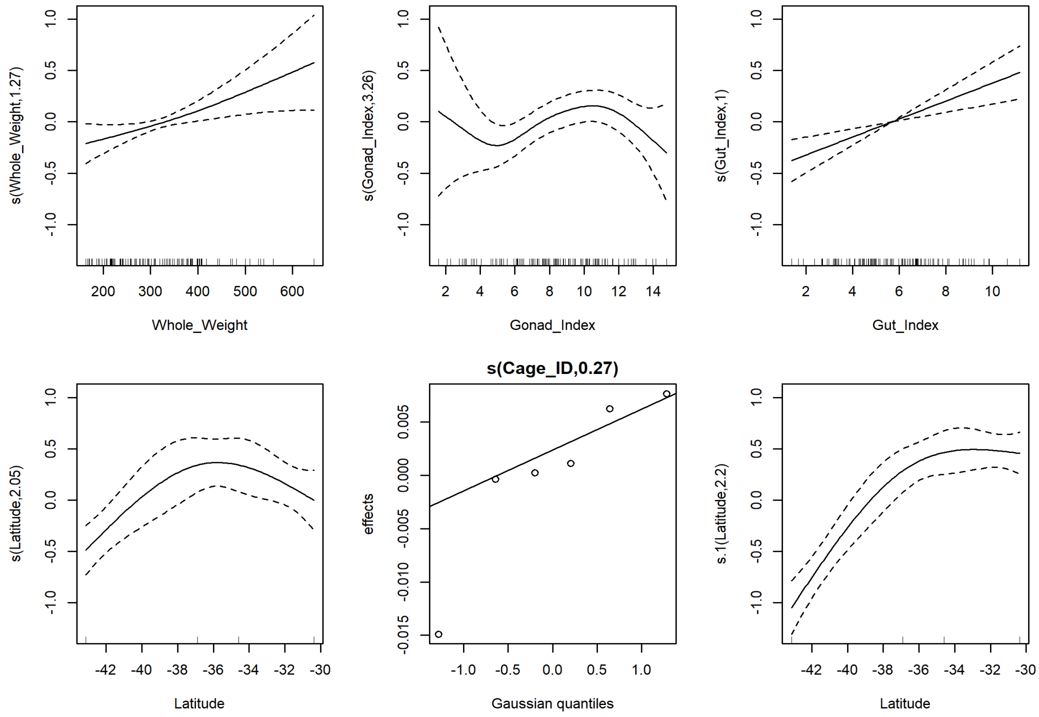

Figure S8: Partial effects plots from the GAM fit to the individual grazing rates of the sea urchin *C. rodgersii* across latitude, and which includes whole wet weight as a predictor. This model shows similar trends and significance of parameters to the mass-independent model.

Table S7: Summary output from the GAM fit to the individual grazing rates of the sea urchin *C. rodgersii* across latitude, and which includes whole wet weight as a predictor. This model shows similar trends and significance of parameters to the mass-independent model.

Component	Term	Estimate	Std Error	t-value	p-value	
A. parametric coefficients	(Intercept)	0.798	0.088	9.092	0.0000	***
	(Intercept).1	-0.374	0.071	-5.236	0.0000	***
**Component**	**Term**	**edf**	**Ref. df**	**F-value**	**p-value**	
B. smooth terms	s(Whole_Weight)	1.268	1.483	8.556	0.0237	*
	s(Gonad_Index)	3.264	4.042	8.699	0.0774	.
	s(Gut_Index)	1.000	1.001	13.748	0.0002	***
	s(Latitude)	2.052	2.278	22.598	0.0000	***
	s(Cage_ID)	0.275	5.000	0.431	0.1556	
	s.1(Latitude)	2.201	2.492	65.667	0.0000	***

Signif. codes: 0 <= '***' < 0.001 < '**' < 0.01 < '*' < 0.05

Adjusted R-squared: NA, Deviance explained 0.526

-REML : 135.589, Scale est: 1.000, N: 107

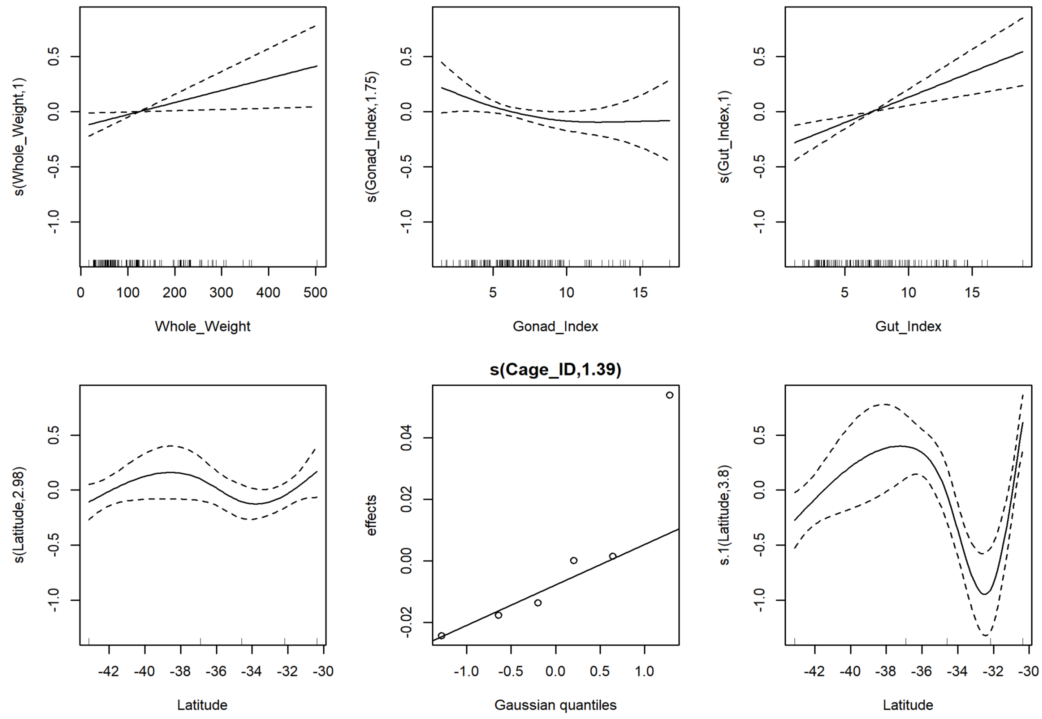

Figure S9: Partial effects plots from the GAM fit to the individual grazing rates of the sea urchin *H. erythrogramma* across latitude, and which includes whole wet weight as a predictor. This model shows similar trends and significance of parameters to the mass-independent model.

Table S8: Summary output from the GAM fit to the individual grazing rates of the sea urchin *H. erythrogramma* across latitude, and which includes whole wet weight as a predictor. This model shows similar trends and significance of parameters to the mass-independent model.

Component	Term	Estimate	Std Error	t-value	p-value	
A. parametric coefficients	(Intercept)	0.247	0.044	5.558	0.0000	***
	(Intercept).1	-0.992	0.068	-14.554	0.0000	***
**Component**	**Term**	**edf**	**Ref. df**	**F-value**	**p-value**	
B. smooth terms	s(Whole_Weight)	1.000	1.000	4.994	0.0254	*
	s(Gonad_Index)	1.749	2.183	4.653	0.1148	
	s(Gut_Index)	1.000	1.000	12.512	0.0004	***
	s(Latitude)	2.976	3.462	6.778	0.1867	
	s(Cage_ID)	1.390	5.000	2.326	0.1377	
	s.1(Latitude)	3.802	3.967	50.859	0.0000	***

Signif. codes: 0 <= '***' < 0.001 < '**' < 0.01 < '*' < 0.05

Adjusted R-squared: NA, Deviance explained 0.325

-REML : 84.648, Scale est: 1.000, N: 120

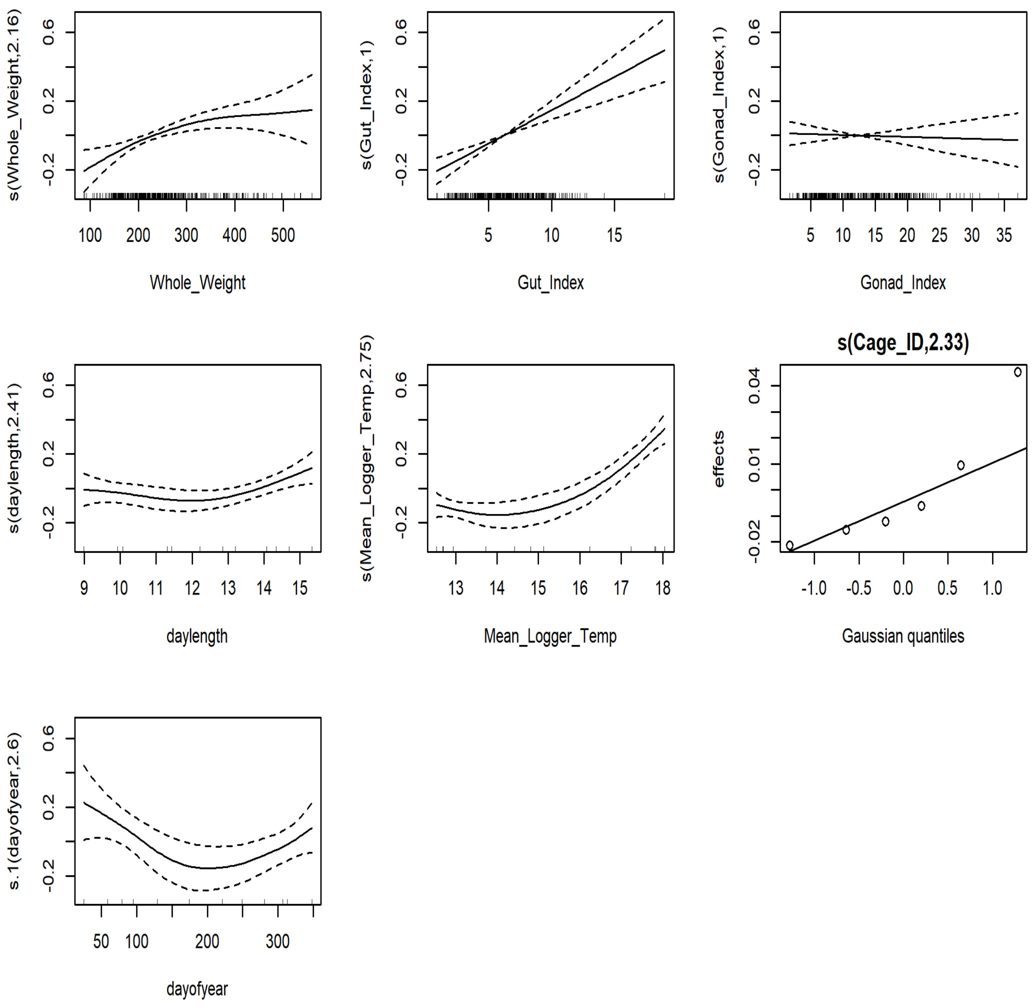

Figure S10: Partial effects plots from the GAM fit to the individual grazing rates of the sea urchin *C. rodgersii* across seasons, and which includes whole wet weight as a predictor. This model shows similar trends and significance of parameters to the mass-independent model.

Table S9: Summary output from the GAM fit to the individual grazing rates of the sea urchin *C. rodgersii* across seasons, and which includes whole wet weight as a predictor. This model shows similar trends and significance of parameters to the mass-independent model.

Component	Term	Estimate	Std Error	t-value	p-value	
A. parametric coefficients	(Intercept)	0.514	0.142	3.625	0.0003	***
	Duration_Exposure	-0.031	0.021	-1.506	0.1322	
	(Intercept).1	-1.268	0.039	-32.779	0.0000	***
**Component**	**Term**	**edf**	**Ref. df**	**F-value**	**p-value**	
B. smooth terms	s(Whole_Weight)	2.159	2.725	19.080	0.0003	***
	s(Gut_Index)	1.000	1.000	29.324	0.0000	***
	s(Gonad_Index)	1.000	1.000	0.118	0.7314	
	s(daylength)	2.408	2.935	7.714	0.0570	.
	s(Mean_Logger_Temp)	2.750	3.307	88.394	0.0000	***
	s(Cage_ID)	2.334	5.000	4.644	0.0745	.
	s.1(dayofyear)	2.599	3.222	8.769	0.0396	*

Signif. codes: 0 <= '***' < 0.001 < '**' < 0.01 < '*' < 0.05

Adjusted R-squared: NA, Deviance explained 0.351

-REML : 106.389, Scale est: 1.000, N: 365

Table S10: Summary output from the GAM fit to the individual grazing rates of the sea urchin *H. erythrogramma* across seasons, and which includes whole wet weight as a predictor. This model shows similar trends and significance of parameters to the mass-independent model.

Component	Term	Estimate	Std Error	t-value	p-value	
A. parametric coefficients	(Intercept)	0.337	0.100	3.376	0.0007	***
	Duration_Exposure	-0.018	0.015	-1.258	0.2085	
	(Intercept).1	-1.394	0.040	-35.234	0.0000	***
**Component**	**Term**	**edf**	**Ref. df**	**F-value**	**p-value**	
B. smooth terms	s(Whole_Weight)	2.775	3.472	14.965	0.0033	**
	s(Gut_Index)	2.446	3.090	45.063	0.0000	***
	s(Mean_Logger_Temp)	1.000	1.000	0.243	0.6220	
	te(Gonad_Index,Mean_Nitrogen)	14.171	16.885	77.137	0.0000	***
	s(Cage_ID)	0.000	6.000	0.000	0.8289	
	s.1(dayofyear)	7.699	8.529	80.547	0.0000	***

Signif. codes: 0 <= '***' < 0.001 < '**' < 0.01 < '*' < 0.05

Adjusted R-squared: NA, Deviance explained 0.351

-REML : 88.994, Scale est: 1.000, N: 363

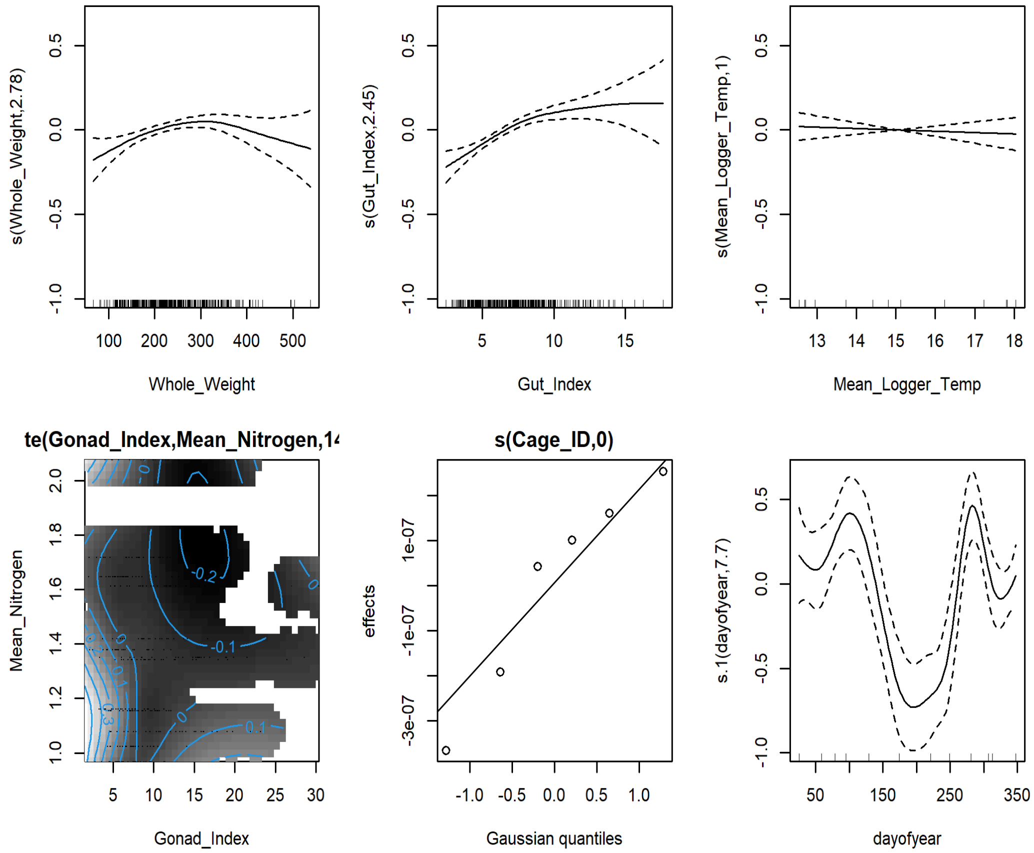

Figure S11: Partial effects plots from the GAM fit to the individual grazing rates of the sea urchin *H. erythrogramma* across seasons, and which includes whole wet weight as a predictor. This model shows similar trends and significance of parameters to the mass-independent model.

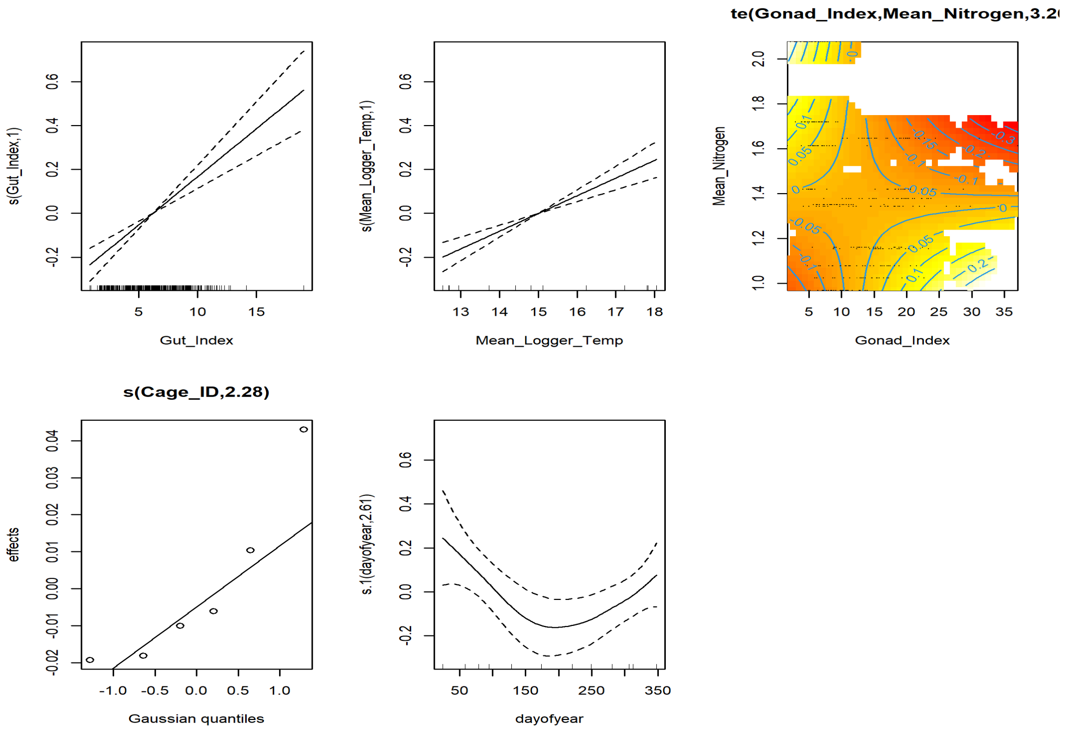

Figure S12: Partial effects plots for a model with similar AIC to the best-fit model which tested potential drivers of seasonal grazing rates of the sea urchin *C. rodgersii*. Both models included gut index, cage ID and the standard deviation for day of year. Compared to the best-fit model, which in addition included temperature and day length, this model instead includes temperature and an interaction between gonad index and nitrogen content of *E. radiata*.

Table S11: Summary output for a model with similar AIC to the best-fit model which tested potential drivers of seasonal grazing rates of the sea urchin *C. rodgersii*. Both models included gut index, cage ID and the standard deviation for day of year. Compared to the best-fit model, which in addition included temperature and day length, this model instead includes temperature and an interaction between gonad index and nitrogen content of *E. radiata*.

Component	Term	Estimate	Std Error	t-value	p-value	
A. parametric coefficients	(Intercept)	0.653	0.142	4.615	0.0000	***
	Duration_Exposure	-0.098	0.021	-4.752	0.0000	***
	(Intercept).1	-1.262	0.039	-32.733	0.0000	***
**Component**	**Term**	**edf**	**Ref. df**	**F-value**	**p-value**	
B. smooth terms	s(Gut_Index)	1.000	1.000	39.101	0.0000	***
	s(Mean_Logger_Temp)	1.000	1.000	36.012	0.0000	***
	te(Gonad_Index,Mean_Nitrogen)	3.262	3.507	16.750	0.0013	**
	s(Cage_ID)	2.278	5.000	4.374	0.0859	.
	s.1(dayofyear)	2.612	3.242	9.339	0.0309	*

Signif. codes: 0 <= '***' < 0.001 < '**' < 0.01 < '*' < 0.05

Adjusted R-squared: NA, Deviance explained 0.323

-REML : 94.962, Scale est: 1.000, N: 365

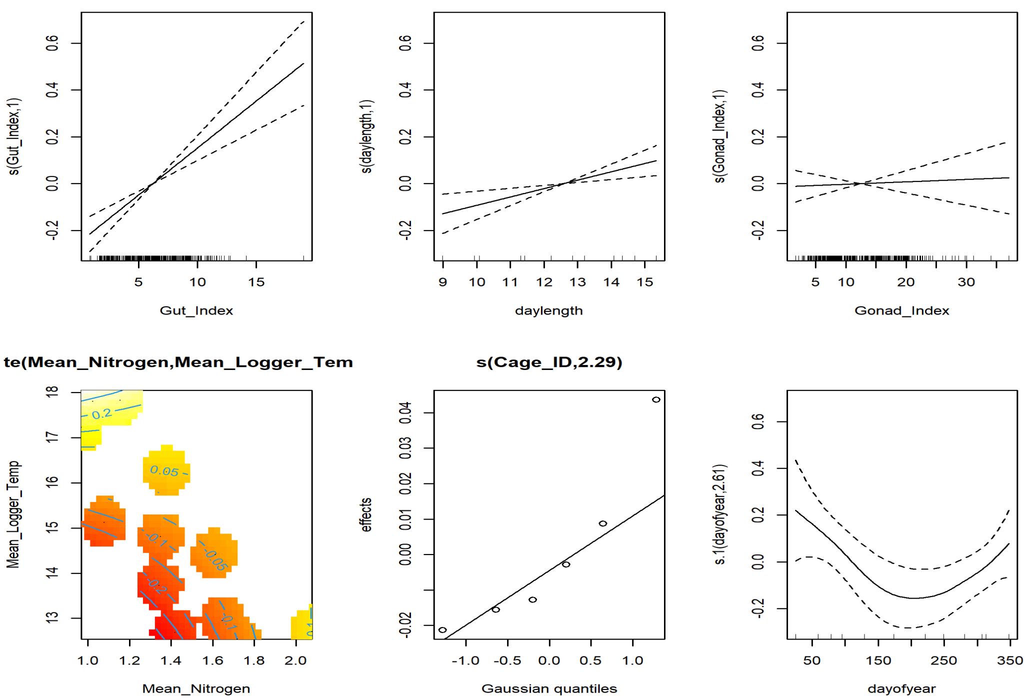

Figure S13: Partial effects plots for a model with similar AIC to the best-fit model which tested potential drivers of seasonal grazing rates of the sea urchin *C. rodgersii*. Both models included gut index, cage ID and the standard deviation for day of year. Compared to the best-fit model, which in addition included temperature and day length, this model instead includes day length, gonad index and an interaction between temperature and nitrogen.

Table S12: Summary output for a model with similar AIC to the best-fit model which tested potential drivers of seasonal grazing rates of the sea urchin *C. rodgersii*. Both models included gut index, cage ID and the standard deviation for day of year. Compared to the best-fit model, which in addition included temperature and day length, this model instead includes day length, gonad index and an interaction between temperature and nitrogen.

Component	Term	Estimate	Std Error	t-value	p-value	
A. parametric coefficients	(Intercept)	0.620	0.147	4.223	0.0000	***
	Duration_Exposure	-0.093	0.021	-4.329	0.0000	***
	(Intercept).1	-1.265	0.039	-32.822	0.0000	***
**Component**	**Term**	**edf**	**Ref. df**	**F-value**	**p-value**	
B. smooth terms	s(Gut_Index)	1.000	1.000	32.602	0.0000	***
	s(daylength)	1.000	1.000	9.384	0.0022	**
	s(Gonad_Index)	1.000	1.000	0.109	0.7417	
	te(Mean_Nitrogen,Mean_Logger_Temp)	3.000	3.000	98.128	0.0000	***
	s(Cage_ID)	2.294	5.000	4.450	0.0830	.
	s.1(dayofyear)	2.606	3.236	8.966	0.0371	*

Signif. codes: 0 <= '***' < 0.001 < '**' < 0.01 < '*' < 0.05

Adjusted R-squared: NA, Deviance explained 0.328

-REML : 97.515, Scale est: 1.000, N: 365

Table S13: The final models used in each of the latitudinal and seasonal analyses, including transformations used to meet model assumptions.

Analysis	Species	Dependant variable	Model type	Model family	Fixed effects	Random effects (smooth)	Location-scale predictor
Latitudinal	*C. rodgersii*	Mass-ind. grazing rate	gam	gaussian location-scale	gonad index + gut index + latitude	cage ID	latitude
	H. erythrogramma	Mass-ind. grazing rate	gam	gaussian location-scale	gonad index + gut index + latitude	cage ID	latitude
	-	log(SST)	gamm	gaussian	site + day of year*site + corrAR(1)	year	-
	-	Nitrogen	gam	gaussian	latitude	-	-
	-	log(C:N)	gam	gaussian	latitude	-	-
	C. rodgersii	log(abundance)	glm	neg. binomial	latitude + latitude^2	-	-
	H. erythrogramma	log(abundance)	glm	neg. binomial	latitude	-	-
Seasonal	*C. rodgersii*	Mass-ind. grazing rate	gam	gaussian location-scale	gut index + gonad index + day length + temperature + assay duration	cage ID	day of year
	H. erythrogramma	Mass-ind. grazing rate	gam	gaussian location-scale	gut index + temperature + gonad index*nitrogen + assay duration	cage ID	day of year
	-	Nitrogen	gam	gaussian	time	-	-
	-	C:N	gam	gaussian	day of year	-	-

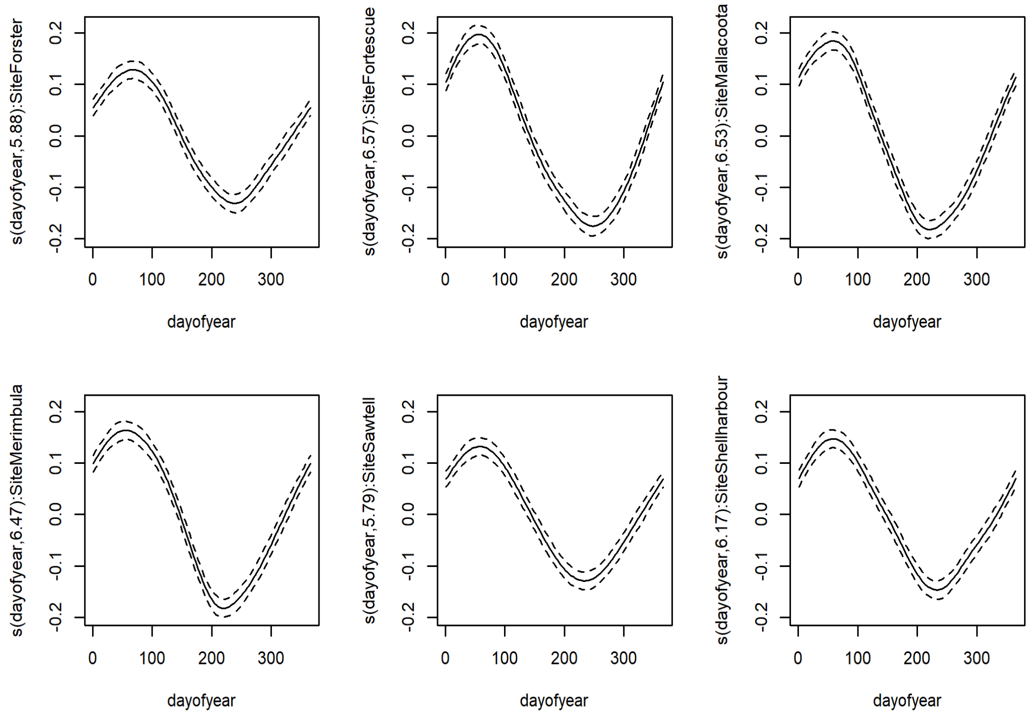

Figure S14: Partial effects plots from the GAMM fit to the annual climatology of sea surface temperature at each of the 6 sites across latitude.

Table S14: Summary output from the GAMM fit to sea surface temperature data.

Component	Term	Estimate	Std Error	t-value	p-value	
A. parametric coefficients	(Intercept)	3.066	0.006	543.344	0.0000	***
	SiteFortescue	-0.359	0.006	-59.756	0.0000	***
	SiteMallacoota	-0.202	0.006	-33.534	0.0000	***
	SiteMerimbula	-0.128	0.006	-21.368	0.0000	***
	SiteSawtell	0.053	0.006	8.897	0.0000	***
	SiteShellharbour	-0.052	0.006	-8.614	0.0000	***
**Component**	**Term**	**edf**	**Ref. df**	**F-value**	**p-value**	
B. smooth terms	s(dayofyear):SiteForster	5.883	8.000	59.561	0.0000	***
	s(dayofyear):SiteFortescue	6.566	8.000	127.831	0.0000	***
	s(dayofyear):SiteMallacoota	6.532	8.000	126.218	0.0000	***
	s(dayofyear):SiteMerimbula	6.467	8.000	110.874	0.0000	***
	s(dayofyear):SiteSawtell	5.790	8.000	61.860	0.0000	***
	s(dayofyear):SiteShellharbour	6.166	8.000	74.104	0.0000	***

Signif. codes: 0 <= '***' < 0.001 < '**' < 0.01 < '*' < 0.05

Adjusted R-squared: 0.929, Deviance explained NA

lme.REML : NA, Scale est: 0.00237, N: 25692

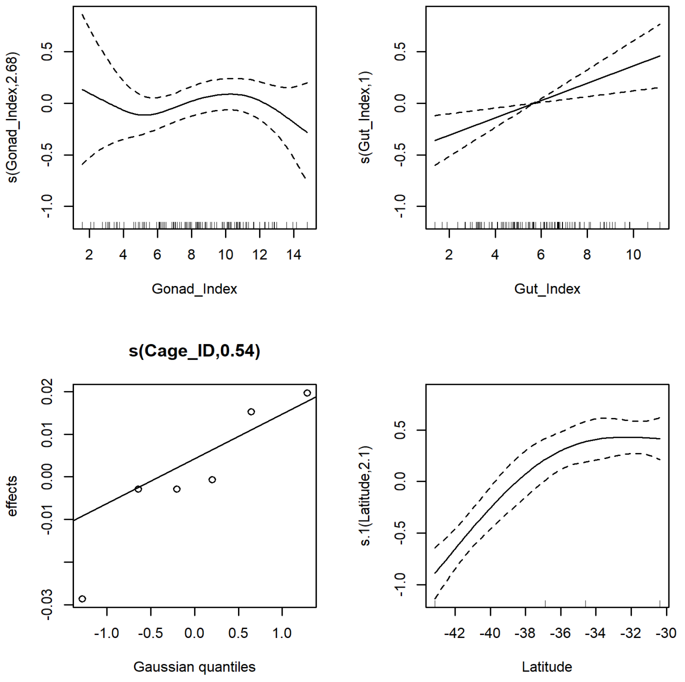

Figure S15: Partial effects plots from the GAM fit to mass-independent grazing rates of the sea urchin *C. rodgersii* across latitude.

Table S15: Summary output from the GAM fit to mass-independent grazing rates of *C. rodgersii* across latitude.

Component	Term	Estimate	Std Error	t-value	p-value	
A. parametric coefficients	(Intercept)	-0.003	0.088	-0.037	0.9707	
	(Intercept).1	-0.329	0.071	-4.639	0.0000	***
**Component**	**Term**	**edf**	**Ref. df**	**F-value**	**p-value**	
B. smooth terms	s(Gonad_Index)	2.680	3.354	4.183	0.3700	
	s(Gut_Index)	1.000	1.000	8.948	0.0028	**
	s(Latitude)	2.069	2.296	17.741	0.0004	***
	s(Cage_ID)	0.543	5.000	0.765	0.2103	
	s.1(Latitude)	2.101	2.389	52.271	0.0000	***

Signif. codes: 0 <= '***' < 0.001 < '**' < 0.01 < '*' < 0.05

Adjusted R-squared: NA, Deviance explained 0.274

-REML : 136.248, Scale est: 1.000, N: 107

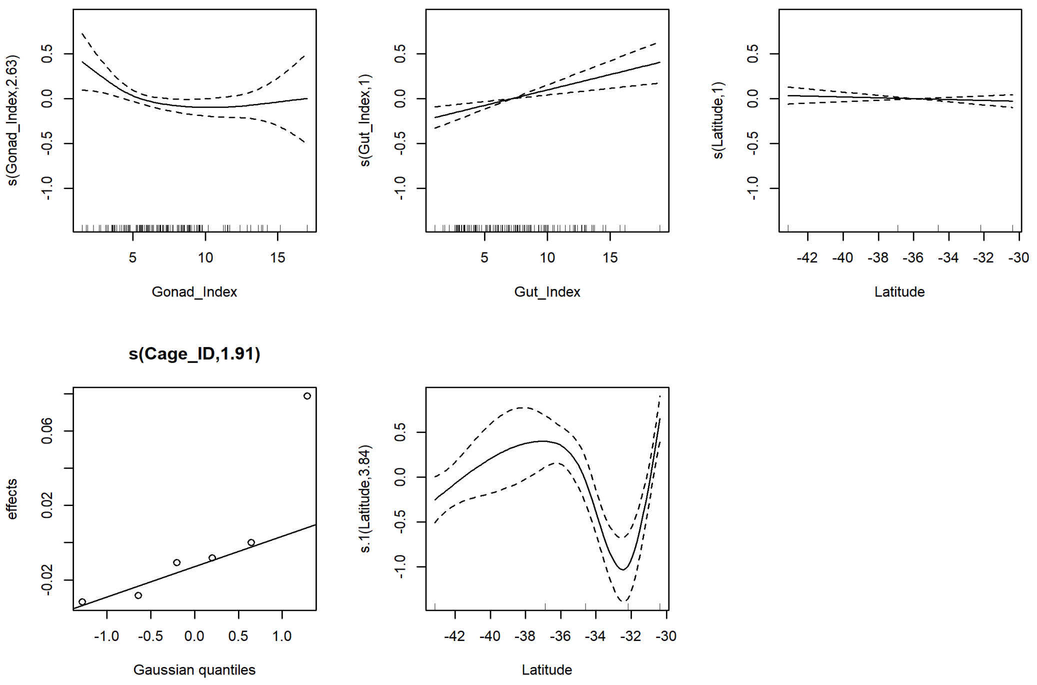

Figure S16: Partial effects plots of the GAM fit to mass-independent grazing rates of the sea urchin *H. erythrogramma* across latitude

Table S16: Summary output from the GAM fit to mass-independent grazing rates of *H. erythrogramma* across latitude.

Component	Term	Estimate	Std Error	t-value	p-value	
A. parametric coefficients	(Intercept)	-0.066	0.042	-1.592	0.1115	
	(Intercept).1	-0.972	0.068	-14.305	0.0000	***
**Component**	**Term**	**edf**	**Ref. df**	**F-value**	**p-value**	
B. smooth terms	s(Gonad_Index)	2.628	3.304	8.103	0.0590	.
	s(Gut_Index)	1.000	1.000	12.352	0.0004	***
	s(Latitude)	1.000	1.000	0.559	0.4549	
	s(Cage_ID)	1.907	5.000	4.262	0.0552	.
	s.1(Latitude)	3.842	3.980	55.724	0.0000	***

Signif. codes: 0 <= '***' < 0.001 < '**' < 0.01 < '*' < 0.05

Adjusted R-squared: NA, Deviance explained 0.321

-REML : 83.554, Scale est: 1.000, N: 120

Table S17: Results of the ANOVA used to compare differences in whole wet weight between sites for *C. rogersii.*

	Sum Sq	Df	F value	Pr(>F)
(Intercept)	913.84	1	16881.86	0.0000
Site	5.13	3	31.60	0.0000
Residuals	5.58	103		

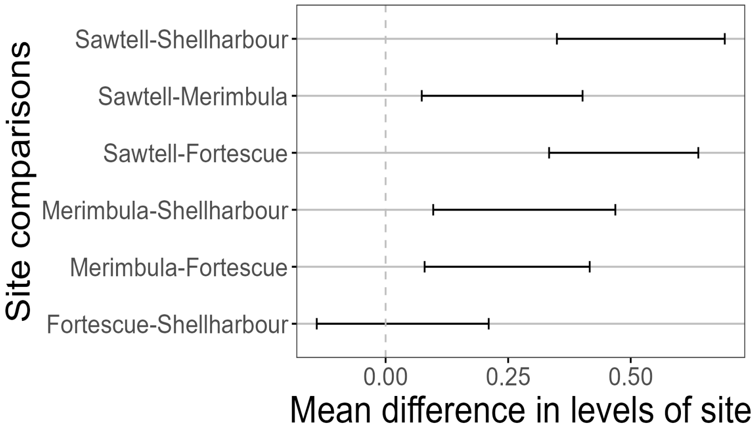

Figure S17: Plotted results from a Tukey HSD showing pairwise site comparisons for whole wet weight of *C. rodgersii.* Where confidence intervals span zero (dashed vertical line) this indicates there is no significant difference between the two sites. Sawtell = site A, Forster = site B, Shellharbour = site C, Merimbula = site D, Forstecue = site E.

Table S18: Results of the ANOVA used to compare differences in whole wet weight between sites for *H. erythrogramma*.

	Sum Sq	Df	F value	Pr(>F)
(Intercept)	370.68	1	1617.56	0.0000
Site	36.53	4	39.85	0.0000
Residuals	26.35	115		

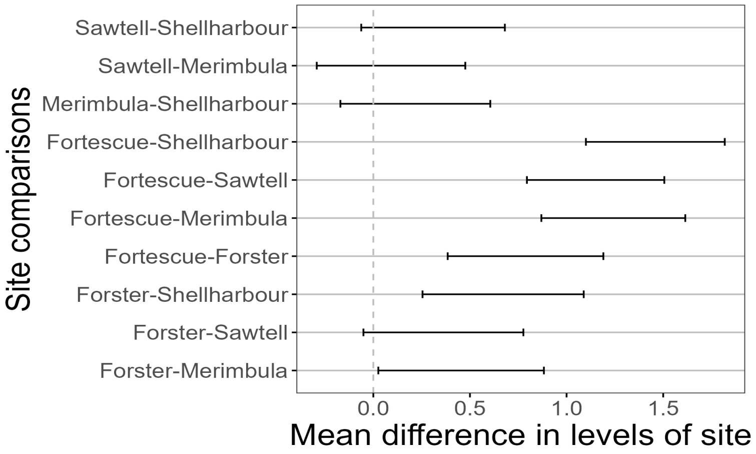

Figure S18: Plotted results from a Tukey HSD showing pairwise site comparisons for whole wet weight of *H. erythrogramma*. Where confidence intervals span zero (dashed vertical line) this indicates there is no significant difference between the two sites. Sawtell = site A, Forster = site B, Shellharbour = site C, Merimbula = site D, Forstecue = site E.

Table S19: The different models fitted to consumption rates across season for each urchin species, and their AIC comparison. Each model includes terms for gut index, duration of assay and cage ID (random effect), in addition to those listed below.

Species	Model terms	df	AIC
C. rodgersii	Temperature + nitrogen + day length+ gonad index	18.4	172.0
	Temperature + nitrogen + gonad index	17.3	177.0
	**Temperature + day length + gonad index**	**17.2**	**170.7**
	Nitrogen + day length + gonad index	17.4	196.8
	Gonad index*temperature + day length	21.7	173.7
	Gonad index*nitrogen + temperature	15.3	170.5
	Temperature*nitrogen + daylength	15.3	168.8
	Temperature*whole weight + day length	23.0	174.3
	Nitrogen*whole weight + temperature	19.2	172.3
H. erythrogramma	Temperature + nitrogen + day length + gonad index	26.1	135.8
	Temperature + nitrogen + gonad index	22.9	136.8
	Temperature + day length + gonad index	24.0	136.6
	Nitrogen + day length + gonad index	25.6	134.2
	Gonad index*temperature + day length	29.8	133.2
	**Gonad index*nitrogen + temperature**	**32.3**	**122.3**
	Temperature*nitrogen + day length	26.9	135.3
	Temperature*Whole weiight + nitrogen	29.7	132.0
	Nitrogen*Whole weight + temperature	29.7	135.9

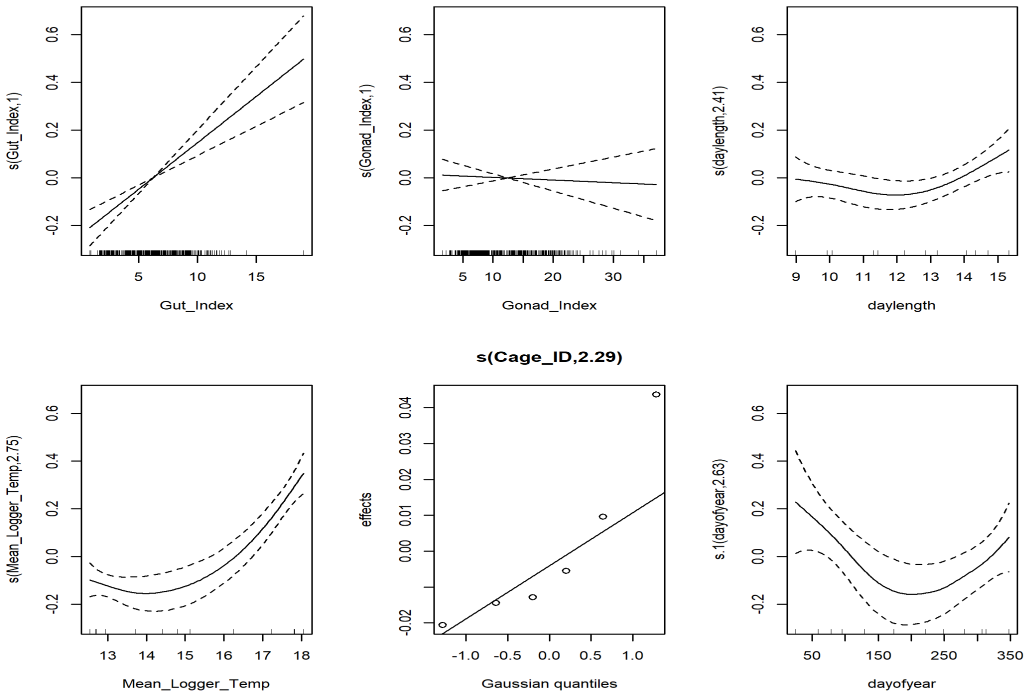

Figure S19: Partial effects plots of the best-fit and simplest GAM for mass-independent seasonal grazing rates of the sea urchin *C. rodgersii*.

Table S20: Summary output from best-fit and simplest GAM for mass-independent grazing rates of *C. rodgersii* in seasonal assays.

Component	Term	Estimate	Std Error	t-value	p-value	
A. parametric coefficients	(Intercept)	0.202	0.141	1.434	0.1516	
	Duration_Exposure	-0.031	0.020	-1.505	0.1324	
	(Intercept).1	-1.267	0.039	-32.803	0.0000	***
**Component**	**Term**	**edf**	**Ref. df**	**F-value**	**p-value**	
B. smooth terms	s(Gut_Index)	1.000	1.000	29.950	0.0000	***
	s(Gonad_Index)	1.000	1.000	0.133	0.7152	
	s(daylength)	2.406	2.933	7.701	0.0577	.
	s(Mean_Logger_Temp)	2.751	3.307	90.106	0.0000	***
	s(Cage_ID)	2.286	5.000	4.441	0.0824	.
	s.1(dayofyear)	2.630	3.261	9.350	0.0319	*

Signif. codes: 0 <= '***' < 0.001 < '**' < 0.01 < '*' < 0.05

Adjusted R-squared: NA, Deviance explained 0.330

-REML : 102.073, Scale est: 1.000, N: 365

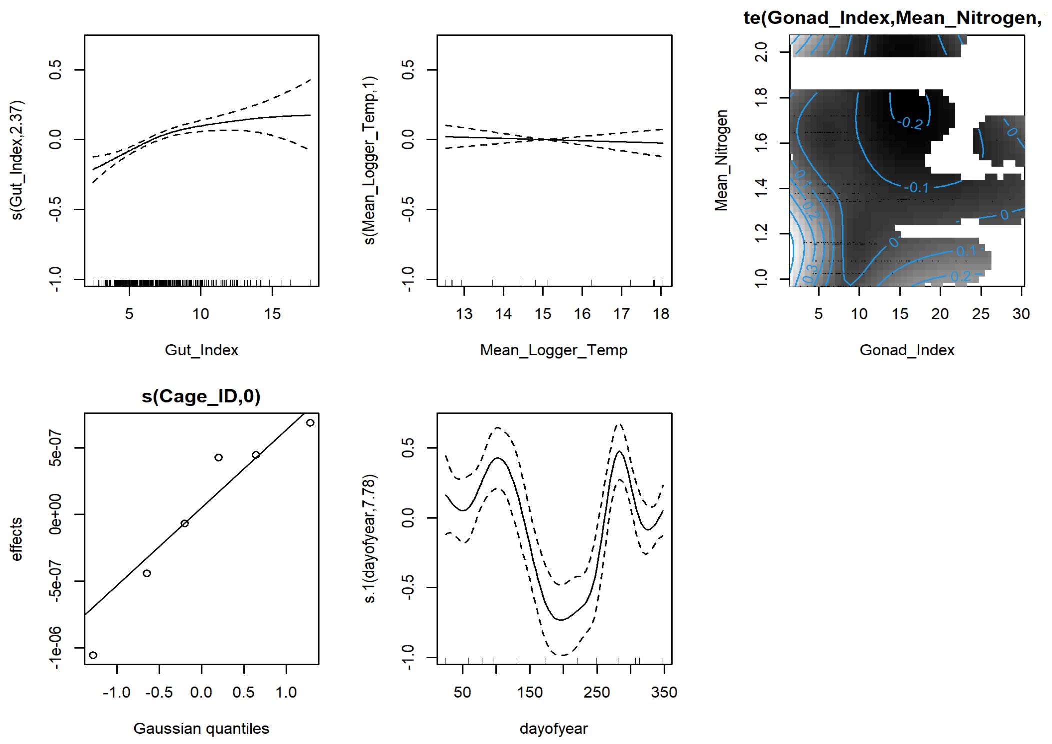

Figure S20: Partial effects plots of the best-fit GAM for mass-independent seasonal grazing rates of the sea urchin *H. erythrogramma*.

Table S21: Summary output from best-fit GAM for mass-indpenedent grazing rates of *H. erythrogramma* in seasonal assays.

**Component**	**Term**	**Estimate**	**Std Error**	**t-value**	**p-value**	
A. parametric coefficients	(Intercept)	0.139	0.099	1.397	0.1623	
	Duration_Exposure	-0.023	0.015	-1.562	0.1182	
	(Intercept).1	-1.380	0.039	-34.998	0.0000	***
**Component**	**Term**	**edf**	**Ref. df**	**F-value**	**p-value**	
B. smooth terms	s(Gut_Index)	2.369	2.997	45.827	0.0000	***
	s(Mean_Logger_Temp)	1.000	1.000	0.255	0.6137	
	te(Gonad_Index,Mean_Nitrogen)	13.996	16.739	72.562	0.0000	***
	s(Cage_ID)	0.000	5.000	0.000	0.6611	
	s.1(dayofyear)	7.775	8.588	82.466	0.0000	***

Signif. codes: 0 <= '***' < 0.001 < '**' < 0.01 < '*' < 0.05

Adjusted R-squared: NA, Deviance explained 0.325

-REML : 87.124, Scale est: 1.000, N: 363

Table S22: Results of the ANOVAs used to compare differences in whole wet weight between temporal sampling events for *C. rogersii*.

	Sum Sq	Df	F value	Pr(>F)
(Intercept)	875.99	1	9081.55	0.0000
factor(Date)	5.46	12	4.72	0.0000
Residuals	33.95	352		

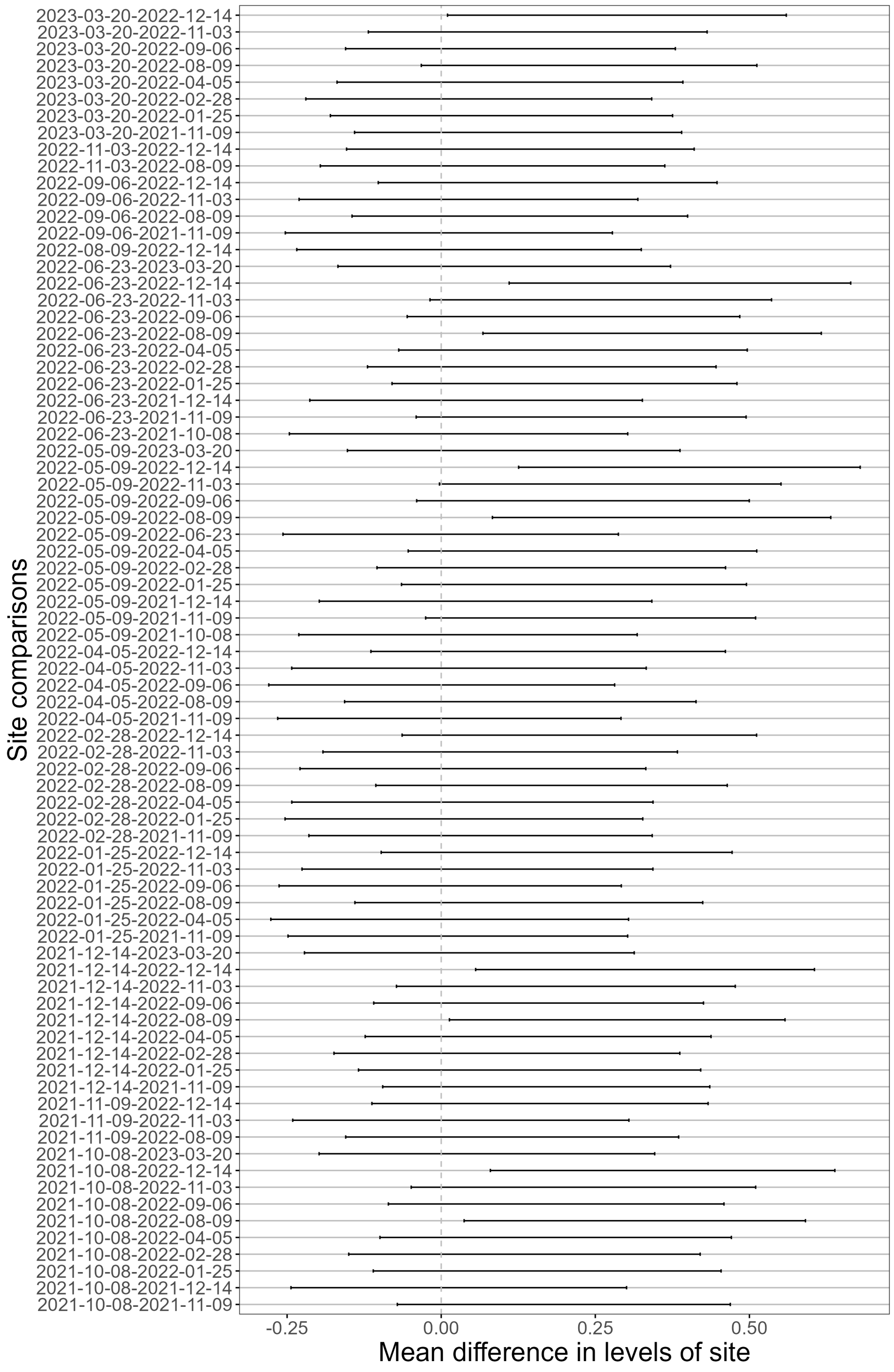

Figure S21: Plotted results from a Tukey HSD showing pairwise comparisons for whole wet weight of *C. rodgersii* across sampling events through time. Where confidence intervals span zero (dashed vertical line) this indicates there is no significant difference between the two sampling periods.

Table S23: Results of the ANOVAs used to compare differences in whole wet weight between temporal sampling events for *H. erythrogramma*.

	Sum Sq	Df	F value	Pr(>F)
(Intercept)	2087694.47	1	354.30	0.0000
factor(Date)	224294.20	12	3.17	0.0003
Residuals	2062333.46	350		

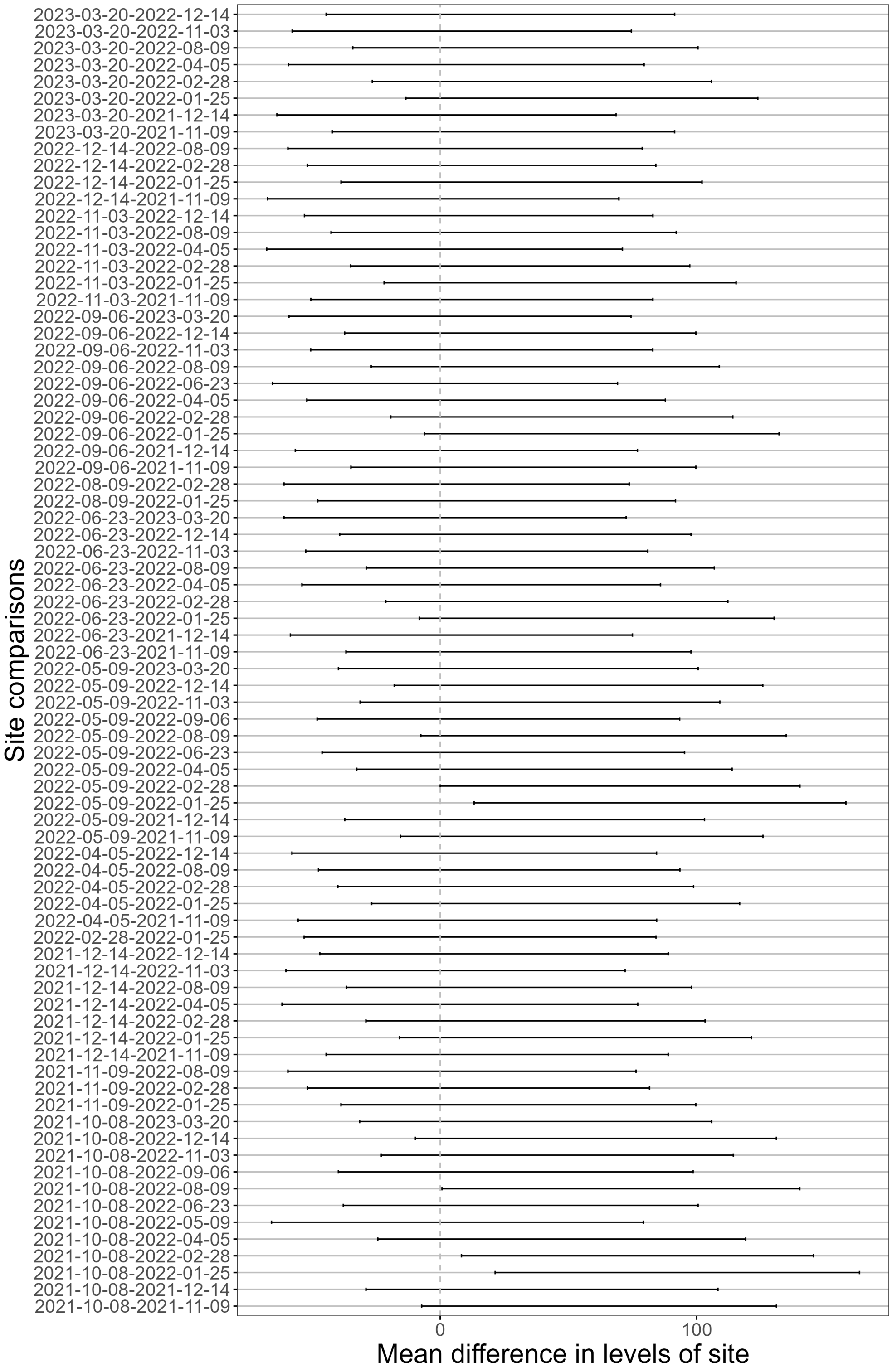

Figure S22: Plotted results from a Tukey HSD showing pairwise comparisons for whole wet weight of *H. erythrogramma* across sampling events through time. Where confidence intervals span zero (dashed vertical line) this indicates there is no significant difference between the two sampling periods.
